# Development and Validation of Tumor-educated Blood Platelets Integrin Alpha 2b (ITGA2B) RNA for Diagnosis and Prognosis of Non-small-cell Lung Cancer through RNA-seq

**DOI:** 10.7150/ijbs.36284

**Published:** 2019-07-24

**Authors:** Shan Xing, Tao Zeng, Ning Xue, Yi He, Yan-zhen Lai, Hui-lan Li, Qi Huang, Shu-lin Chen, Wan-li Liu

**Affiliations:** 1 Department of Clinical Laboratory, State Key Laboratory of Oncology in South China, Collaborative Innovation Center for Cancer Medicine, Sun Yat-sen University Cancer Center, Guangzhou 510060, P. R. China; 2 Department of Clinical Laboratory, The Sixth Affiliated Hospital, Sun Yat-sen University, Guangzhou 510060, P. R. China; 3 Department of Clinical Laboratory, Affiliated Tumor Hospital of Zhengzhou University, Henan Tumor Hospital, Zhengzhou 450100, P. R. China

**Keywords:** NSCLC, diagnosis, survival, tumor-educated blood platelets, ITGA2B

## Abstract

**Background:** Currently, there are no molecular biomarkers for the early detection of non-small-cell lung cancer (NSCLC). This study focused on identifying RNAs found on tumor-educated blood platelets (TEPs) for detecting stage I NSCLC.

**Methods:** Platelet RNAs, isolated from the blood of 9 patients with NSCLC (stages I and II) and 8 healthy controls, were analyzed using RNA-seq. ITGA2B was selected as a candidate marker. Two different Polymerase Chain Reactions (PCR) were used to measure ITGA2B in platelet samples from healthy controls (n = 150), patients with NSCLC (n = 243), and patients with benign pulmonary nodules (n = 141) in two cohorts.

**Results:** Platelet ITGA2B levels were significantly higher (*p* < 0.001) in patients with NSCLC than in all controls. The diagnostic accuracy of ITGA2B was area under the curve (AUC) of 0.922 [95% confidence interval (CI), 0.892-0.952], sensitivity of 92.8%, and specificity of 78.6% in the test cohort and 0.888, 91.2%, and 56.5% in the validation cohort for NSCLC by quantitative real time PCR (q-PCR). Furthermore, ITGA2B maintained diagnostic accuracy for patients with NSCLC using Droplet Digital PCR (ddPCR) and the other type of internal control, Ribosomal Protein L32 (RPL32) [ddPCR: 0.967 (0.929-1.000) and RPL32: 0.847(0.773-0.920)]. A nomogram incorporating ITGA2B, carcinoembryonic antigen (CEA) and stage could predict the overall survival (C-index = 0.756).

**Conclusions:** TEP ITGA2B is a promising marker to improve identification of patients with stage I NSCLC and differentiate malignant from benign lung nodules.

## Introduction

Lung cancer is one of the most common causes of death in the world^1^, ^2^. Non-small cell lung cancer (NSCLC) is the main type of lung cancer which generally consists of two histological types: adenocarcinoma (ADC) and squamous cell carcinoma (SqCC). Like other cancers, the majority of cases (67%) are detected at an advanced stage^3^. Delayed diagnosis results in elevated mortality, NSCLC patients found at stage I or II have a survival rate of 71%, whereas < 2% for the stage IV patients. Low-dose computed tomography (LDCT) screening is developed for the screening of lung cancer in high-risk individuals^4^, ^5^, and this approach has suggested a sensitivity of 93.8% and a reduction in mortality of 20%. However, LDCT lacks the specificity to identify indeterminate pulmonary nodules (IPNs), which results in high rate of false-positive results (up to 96%)^6^. Some of the benign patients received transthoracic needle lung biopsy, which may lead to pneumothorax requiring chest tube^7^, and a high rate (24%) of them undergo nontherapeutic operations^6^. Moreover, repeated computed tomography (CT) scanning may cumulate radiation, thus increases the concern of an elevated risk of developing radiation-related cancer.

The most rationale solution to early detection and improve the specificity of screening is to discover reliable markers which are capable of early detecting and differentiating malignant from benign lung nodules through simple blood tests^8^. CEA is the most common used serum biomarker for cancer screening and the diagnosis of NSCLC, cytokeratin-19 fragment (CYFRA21-1) is also applied in NSCLC detection clinically^9^-^12^. However, the low sensitivity and incremental concentrations in benign diseases hamper their application^3^, ^11^, ^13^-^18^. Reliable and novel diagnostic biomarkers, particularly those shed directly from tumor cells are in urgent need to improve clinical outcomes.

Platelets, the second most-abundant cell type in peripheral blood, have the basic functions in coagulation and haemostasis^19^. Platelets are a fundamental component of the tumor microenvironment and serve as local and systemic respondents during tumor initiation and tumor metastasis^20^, thus being exposed to tumor-mediated platelet education, and leading to changed platelet behavior. TEPs broadened the spectrum of liquid biopsy applications, and may enable blood-based cancer diagnostics^21^. In the aspect of implementation for early detection of cancer, it has been reported that thromboSeq, the highly multiplexed TEPs signature detection platform, may enable blood-based cancer diagnostics^22^, ^23^. However, these reports had some limitations, such as rare early stage patients involved, absence of controls with benign pulmonary nodules, and assessments being done via self-learning support vector machine (SVM)-based algorithms, that means, multiple negative factors including global availability of the technology and cost limits their application in screening. Therefore, we designed a simple PCR based study to assess the TEP mRNA and evaluated their accuracy in early detection of NSCLC. This simple, but accurate test would provide new insights in NSCLC screening.

## Materials and Methods

### Ethical statement

This study was reviewed and approved by the Institutional Review Board and Ethics Committee of Sun Yat-Sen University Cancer Center (SYSUCC), Guangzhou, China (GZR2017-186). For patients treated in our center, as a general standard procedure, we will get their written informed consent for the use of clinical parameters and collected samples for further studies at the time of patients' admission. The records were anonymous and de-identified before use.

### Study design

**Figure 1:**
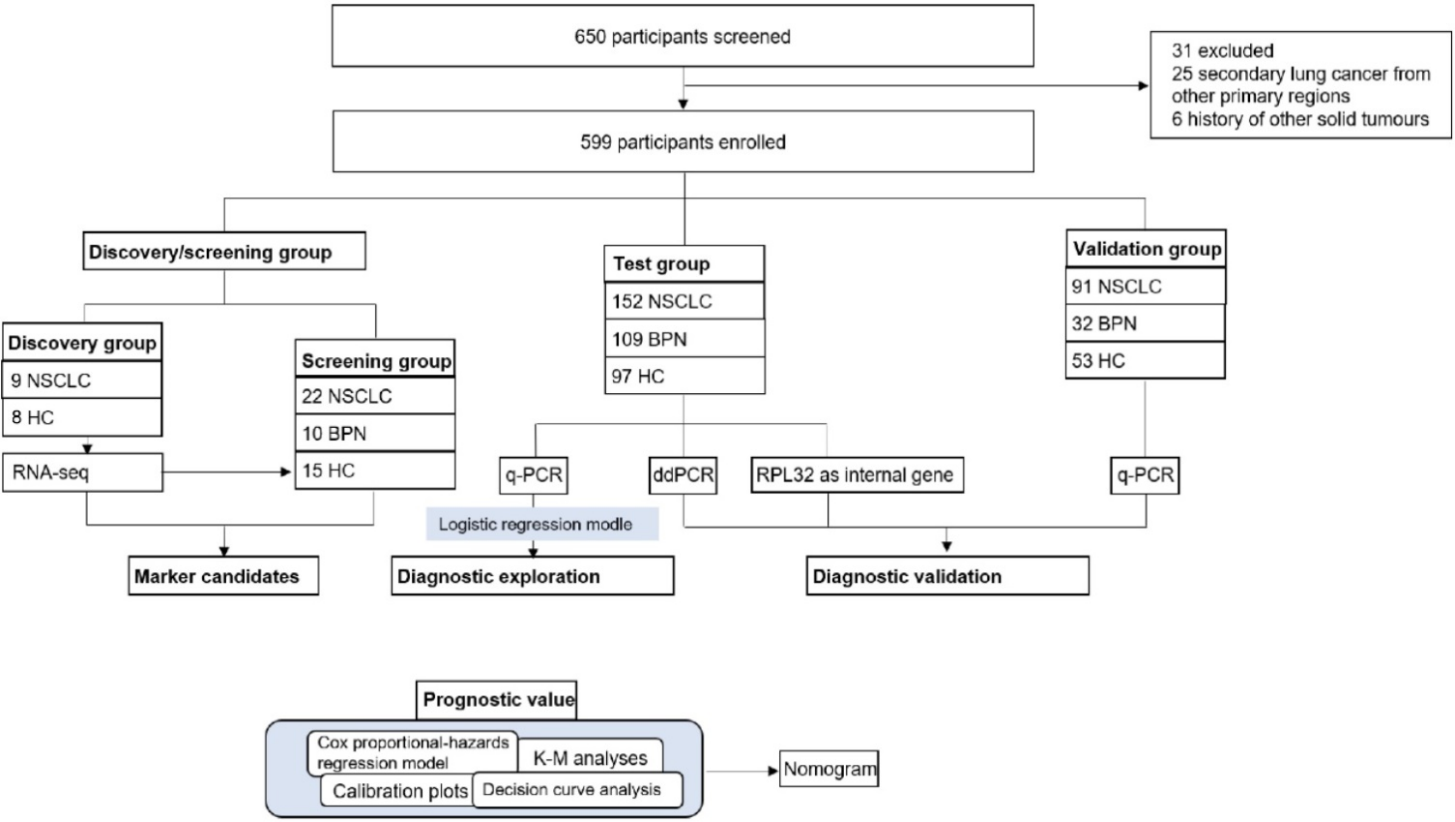
** Study profiles.** NSCLC: non-small cell lung cancer; BPN: benign pulmonary nodules; HC: healthy controls; K-M analyses: Kaplan-Meier analyses; q-PCR: quantitative real-time PCR; ddPCR: Droplet digital PCR

Our study had four progressive stages: candidates selection stage, the test, the validation stage and prognostic analysis, seen in Figure 1. For the initial screening, we performed an RNA-seq and selected potential TEP markers based on expression difference and functionality, and verified their discriminatory power using q-PCR. Next, platelet mRNAs verified were measured in test cohort. Data thus collected were combined with the currently most used screening marker, CEA to yield a diagnostic model. In the validation stage, the diagnostic panel built in the test stage was verified in another independent cohort, an absolute quantification methodology: ddPCR and a different internal control gene, RPL32. At last, we integrated certain TEP mRNA and clinical parameters to create a nomogram, and evaluated the effect of predicting the OS rate of patients with NSCLC.

### Patient cohort and platelets collection

17 platelets from 9 NSCLC (8 stage I and 1 stage Ⅱ) and 8 healthy controls (HC) were collected for RNA-seq. The preliminary screening group included 22 patients with NSCLC, 10 patients with benign pulmonary nodules (BPN), and 15 HC, the details were listed in the supplementary materials. We consecutively enrolled 152 NSCLC patients, 109 BPN, and 97 HC to a test cohort. An independent validation cohort comprising 91 patients with NSCLC, 32 BPN, and 53 HC was recruited. The 108 ddPCR (60 NSCLC, 16 BPN, and 32 HC) and 109 RPL32 (65 NSCLC, 28 BPN, and 16 HC) validation group samples were selected from the test cohort randomly. All the platelet and serum samples were collected at the SYSUCC between 2015 and 2016.

We included the patients with NSCLC with the following criteria: pathologically diagnosed with NSCLC by two independent observers; no history of anti-neoplastic therapy, such as chemotherapy, or radiotherapy; no second carcinoma and the absence of clinical evidences of infection or other inflammation within 1 month, as determined from routine laboratory tests, ultrasonographic examination, CT, and the clinical history. The tumors were staged according to the AJCC 7th edition tumor-node-metastasis (TNM) classification and staging system. After completion of therapy, patients were prospectively followed with physical examination and CT of the chest every 3 months at our outpatient department for the first 2 years, then annually. The last follow-up was on September 11th, 2018.

All selected patients with BPN were pathologically diagnosed or confirmed using CT, and subsequently monitored for more than 2 years via CT screening without evidence of nodule growth, clinical diagnosis of cancer and a history of previous cancer or chemotherapy. None of the enrolled healthy controls suffered from pulmonary diseases, any inflammatory diseases or other diseases, according to a physical examination, including routine laboratory tests, ultrasonographic examination, and CT.

Platelets were isolated from 2ml whole blood in purple-cap BD Vacutainers containing EDTA anti-coagulant. To minimize effects of long-term storage of platelets at room temperature and loss of platelet RNA quality and quantity, samples were processed within 24 hours after blood collection. Samples for both test and validation cohort were collected and processed similarly and simultaneously.

Collected blood samples were centrifuged at 100g for 20min to obtain platelet-rich plasma (PRP). Subsequently, the plasma was centrifuged at 10,000g for 20min. The platelet pellet was resuspended in 30μL RNAlater (LifeTechnologies, Carlsbad, CA) and frozen at -80°C for subsequent use. Platelet purity and quantity was verified by morphological observation of randomly selected and the Sysmex XN2000 haematology analyser with both the sheath flow direct current (DC) detection and PLT-fluorescent method.

Serum samples were centrifuged at 3000g for 10min and stored at -80°C until use. The concentrations of CEA and CYFRA21-1 in the serum were measured using electrochemiluminescence immunoassay (ECLIA) kits on a Roche E602 (Roche, German).

### RNA-seq analysis

1 mL Trizol reagent (Invitrogen, USA) was added to the 17 platelets. RNA was extracted following the Trizol reagent manual. RNA was precipitated in 1:1 isopropanol (v/v) and 1μL glycogen at -20°C overnight. mRNA library was constructed using MGIEasy mRNA library kit following the manufacturer's instructions. Libraries were sequenced on an MGI-seq-2000 sequencer for 50 cycles. Reads passed the MGI quality filters were kept for the subsequent analyses. Adapters were trimmed from the reads, and the reads shorter than 17 nt were discarded. The reads were mapped to the human mRNA reference database using FANSe3 algorithm^24^ on Chi-Cloud NGS Analysis Platform (Chi-Biotech Co. Ltd., Shenzhen, China). The quantity of gene expression was calculated by the RPKM method (Reads Per Kb per Million reads). The genes with FDR less than 0.01 and change fold more than 2 were considered as the DEGs. Gene Ontology (GO) and Kyoto Encyclopedia of Genes and Genomes (KEGG) pathway analysis were conducted by topGO (version 2.18.0) (http://www.bioconductor.org/packages/release/bioc/html/topGO.html) and KOBAS (kobas3.0)^25^, respectively. DEG-GO-pathway correlation network was established through Cytoscape (http://www.cytoscape.org).

### Quantitative real time PCR (q-PCR) and Droplet digital PCR (ddPCR)

Total RNA was extracted following the TRIzol reagent manual and reverse trascribed with the SuperScript II reverse transcriptase (Invitrogen, USA). β-actin was used as an internal control, and RPL32 was also applied in the validation stage. q-PCR was done with a SYBR green-based assay in a LightCycler® 480 II system (Roche, German). The cycling conditions were 5 min at 95°C, 40 cycles of 10 s at 95°C, 10 s at 57°C, 10 s at 72°C, with a final extension of 10 min at 72°C. Relative expression levels of mRNA were calculated based on the threshold cycle (Ct) values, corrected by β-actin expression, with the 2^-ΔCt^ equation. The following primers were used: ITGA2B (GenBank NM_000419.4) forward primer: 5'-CTTTGACCTCCGTGATGAGACC-3'; reverse primer: 5'-CAGTCTTTTCTAGGACGTTCCAGTG-3'; SELP (GenBank NM_003005.3) forward primer: 5'-TGGCAAGTGGAATGATGAGC-3'; reverse primer: 5'-GCAGGTGTAGTTCCCGATGG-3'; β-actin (ACTB, GenBank NM_001101.3) forward primer: 5'-CTGGAACGGTGAAGGTGACA-3', reverse primer: 5'-AAGGGACTTCCTGTAACAATGCA-3', RPL32 (RPL32, GenBank NM_000994.3) forward primer: 5'-TCAAGGAGCTGGAAGTGCTG-3', reverse primer: 5'-CATTGGGGTTGGTGACTCTG-3', more primers were shown in the supplementary. Ten microliters real-time PCR reaction system containing 4μl diluted cDNA product (1:5 in H_2_O), 5μl SYBR green mix (Roche, German), 1μl diluted primer mix (1:40 in H_2_O). All the experiments were done in triplicate. β-actin CT value ≥ 30 samples were excluded in our study as their low RNA concentration.

DdPCR was performed on QX200(Bio-Rad, USA) in an EvaGreen program. The reaction mixture was prepared with a 10μL 2×QX200 ddPCR EvaGreen supermix, 8μl cDNA, 2μl diluted primer mix (1:40 in H_2_O) in a final volume of 20μl. Each reaction load into Cartridge followed by 70μL of oil into the wells. After droplet generation with the QX200 Droplet generator, transfer droplets into a 96-well plate. The plate was sealed with foil and proceed to thermal cycling in an Eppendorf thermocycler, and an initial enzyme activation step (95°C, 5 min) was followed by 40 cycles of 95°C for 15 s and 60°C for 1 min. Signal stablization was 4°C for 5 min and 90°C for 5 min. After thermal cycling, place the plate in the QX200 Droplet Reader. The data were analyzed using absolute quantification according to the manufactures' instruction. β-actin quantitation was diluted by 100 fold because copy number higher than the limit of detection. All samples droplets events > 10000 were further diluted and confirmed in the ddPCR assay. Both of data were normalized to β-actin to determine the relative mRNA levels in platelets. The coefficient of variation (CV) of intra-assay and inter-assay of both PCR assay was less than 12%.

### Statistical analysis

Statistical analyses were performed with the SPSS 23.0 (SPSS Inc.) and R version 3.4.0 (http://www.R-project.org/). Differences between NSCLC group and control were tested with the Mann-Whitney U test. Receiver operating characteristics (ROC) curves were established to evaluate respective AUCs, sensitivity, and specificity. We considered the value with the maximization of the Yuden index as the cut-off value. To combine the biomarkers and assess their complementary effect, a new variable was estimated to predict the probability (*p*) for NSCLC on the basis of an equation obtained by binary logistic regression (all NSCLC versus all controls). The correlation between TEP ITGA2B and SELP mRNA and clinicopathological characteristics was analyzed with Pearson's χ2 test or Continuity Correction. The Spearman's rank correlation coefficient was used to compare the relationship between ITGA2B and SELP mRNA and the results generated by the two PCR methods and two internal control. Medcalc were used to compare the ROC of CEA, ITGA2B and their combination. The duration of OS was calculated from diagnosis of malignancy until study completion or until death. We used the Kaplan-Meier method to evaluate OS and calculate the 95% CIs. To estimate the survival difference, the univariate and multivariate analyses were executed using the Cox proportional hazards model and were shown as hazard ratios (HRs) and 95% CIs. All variables in the multivariable model were enrolled to construct a prognostic nomogram model by the rms package. Calibration of the nomogram for 1- and 2-year OS were executed by comparing the predicted survival and observed survival. The discriminative ability and predictive accuracy of the established nomogram were assessed by C-index and decision curve, and were compared with the traditional TNM staging system, CEA, or ITGA2B. All statistical tests were two-sided, and we consider *p* < 0.05 as statistically significant.

## Results

### The preliminary screening phase

**Figure 2:**
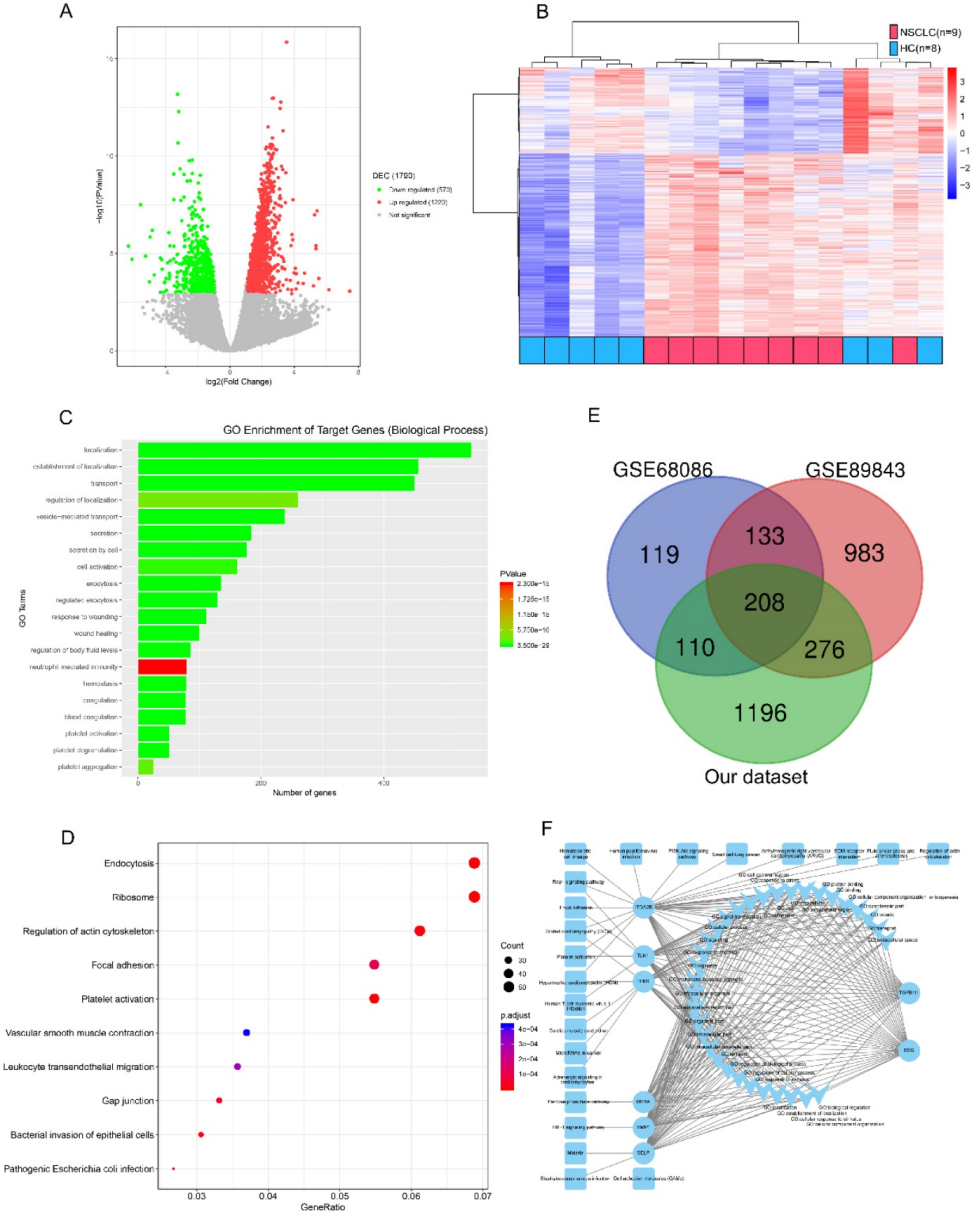
** Candidate platelet mRNA selection from RNA-seq analysis. (A)** Volcano plot of mRNAs expression in 9 early stage NSCLC versus 8 HC. **(B)** Top 20 enrichment of GOs for differentially expressed mRNAs in NSCLC. Red for NSCLC, and blue for HC. **(C)** Bubble plot of top 10 enrichment of Pathways for differentially expressed mRNAs in NSCLC. **(D)** Venn diagram analysis of differentially expressed mRNAs from our dataset, GSE68086 and GSE89843. **(E)** mRNA-GO-pathway correlation network analysis of core mRNAs and their functions. NSCLC: non-small cell lung cancer; HC: healthy controls

We refined the candidates from the RNA-seq DEGs as Figure 1 and Table S1 shown. There were 1220 upregulated and 570 downregulated DEGs based on an analysis of the RNA transcriptomes of 9 patients with early NSCLC and 8 healthy controls (Figure 2A). Unsupervised hierarchical clustering based on the DEGs distinguished NSCLC from HC with minor overlap (Figure 2B). GO analysis revealed that the increased TEP mRNAs were enriched for biological processes such as location, transport and platelet related (Figure 2C) while decreased mRNAs were strongly involved in RNA processing, generally consistent with GSE68086^22^ and GSE89843^23^ enriched. The top enriched KEGG pathways were endocytosis, adhesion and platelet activation (Figure 2D). To guarantee the reproducibility, we selected the 208 overlapped DEGs in our dataset, GSE68086 and GSE89843 (Figure 2E). Because upregulated makers are easier to assess and most of the clinically used traditional tumor markers were upregulated in cancer patients, we focused on the 148 upregulated DEGs. Markers for screening and early detection should take sensitivity into first account, 119 TEP mRNAs upregulated in at least 7 out of 9 NSCLC samples were selected. And the 104 TEP mRNAs with mean Reads Per Kilo bases per Million reads (RPKM) more than 20 from the 9 NSCLC samples were enrolled considering abundant TEP mRNAs in NSCLC were easier to access thus conductive to screening. As TEPs contribute to the spread of cancer, 8 differentially detected platelet mRNAs are noteworthy. They may be involved in important biological processes or signaling pathways in tumors, according to the DEG-GO-KEGG pathway network and literatures (Figure 2F). They are BSG^26^, DERA^27^, ITGA2B^28^, SELP^29^, TGFB1I1^30^, TIMP1^31^, TLN1^32^, and TPM1^33^. We also randomly selected two genes without significant regulation, CD63 and IFITM3 as negative controls. These 10 biomarkers were subjected to an q-PCR assay using platelets from 22 NSCLC patients and 25 controls (10 patients with BPN and 15 HC). Platelet purity and quantity was confirmed (contamination is 1 to 5 nucleated cells per 10 million platelets, see Figure S1). As shown in Figure S2, the negative controls, CD63 and IFITM3 indeed displayed poor discriminatory effect. The mRNA levels of platelet ITGA2B (*p* < 0.001), and SELP (*p* < 0.001) were significantly elevated in the NSCLC patients compared with either of the two controls, respectively. TGFB1I1, DERA, TIMP1 and TPM1 exhibited higher levels only when the NSCLC patients group compared with benign nodules group but not the healthy group. Besides the levels of BSG were significantly higher in the NSCLC patients compared with healthy controls instead of BPN. Whereas TLN1 provided poor discriminatory performance. We studied the diagnostic effect of ITGA2B and SELP in subsequent analyses.

### Performance of 2 markers in the test and validation cohort

To verify the discriminatory capability of platelet ITGA2B and SELP, we utilized platelets from 534 participants overall, including 358 in the test cohort and 176 in the validation cohort. Clinicopathological parameters of NSCLC patients in the two cohorts were present in the Table 1. The cohorts were well matched for age, sex, smoking status, histology type and stage overall, except that fewer proportion patients with adenocarcinoma were enrolled into the validation cohort than into the test cohort (*p* = 0.049). Here we compared the traditional tumor markers, CEA and CYFRA21-1 in a subgroup of the test cohort, and found that CEA performed better in the diagnosis of NSCLC (See in the supplement materials and Figure S3).

**Figure 3:**
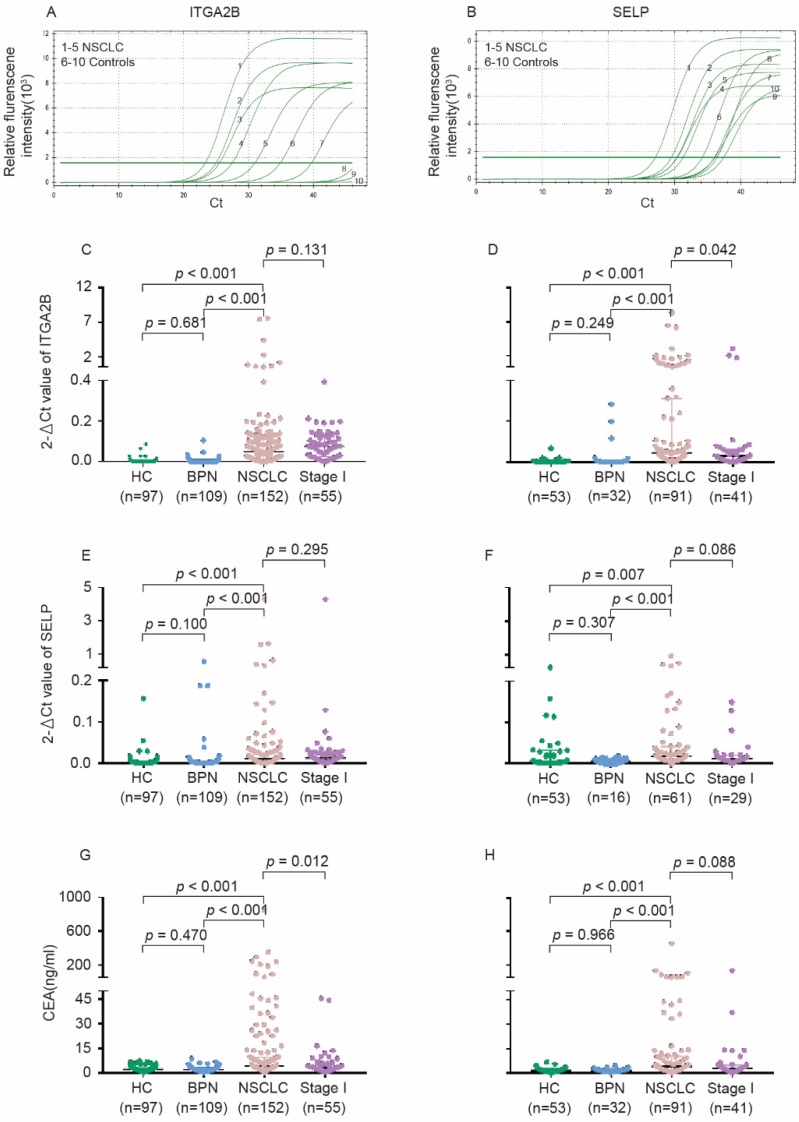
** Levels of platelet ITGA2B, SELP mRNA and serum CEA concentrations in the test and validation cohorts.** The representative amplification curve of Real-time PCR reaction for ITGA2B (A) and SELP (B). Levels of ITGA2B for test cohort (C). ITGA2B for validation cohort (D). SELP for test cohort (E). SELP for validation cohort (F). CEA for test cohort (G). CEA for validation cohort (H). NSCLC: non-small cell lung cancer; BPN: benign pulmonary nodules; HC: healthy controls; ITGA2B: Integrin, Alpha 2b; SELP: P-selectin; CEA: carcinoembryonic antigen.

As present in Figure 3 and Table S2, the relative expression levels of platelet ITGA2B and SELP mRNA on q-PCR were dramatically higher in NSCLC patients than in all controls (*p* < 0.001); However, values did not differ significantly between the two control groups. The levels of SELP were significantly associated with ITGA2B (*p* < 0.001). Likewise, the median concentration of CEA in serum showed the similar tendency. However, the protein levels of platelet ITGA2B and SELP were likely similar in NSCLC and controls (Figure S4).

**Figure 4:**
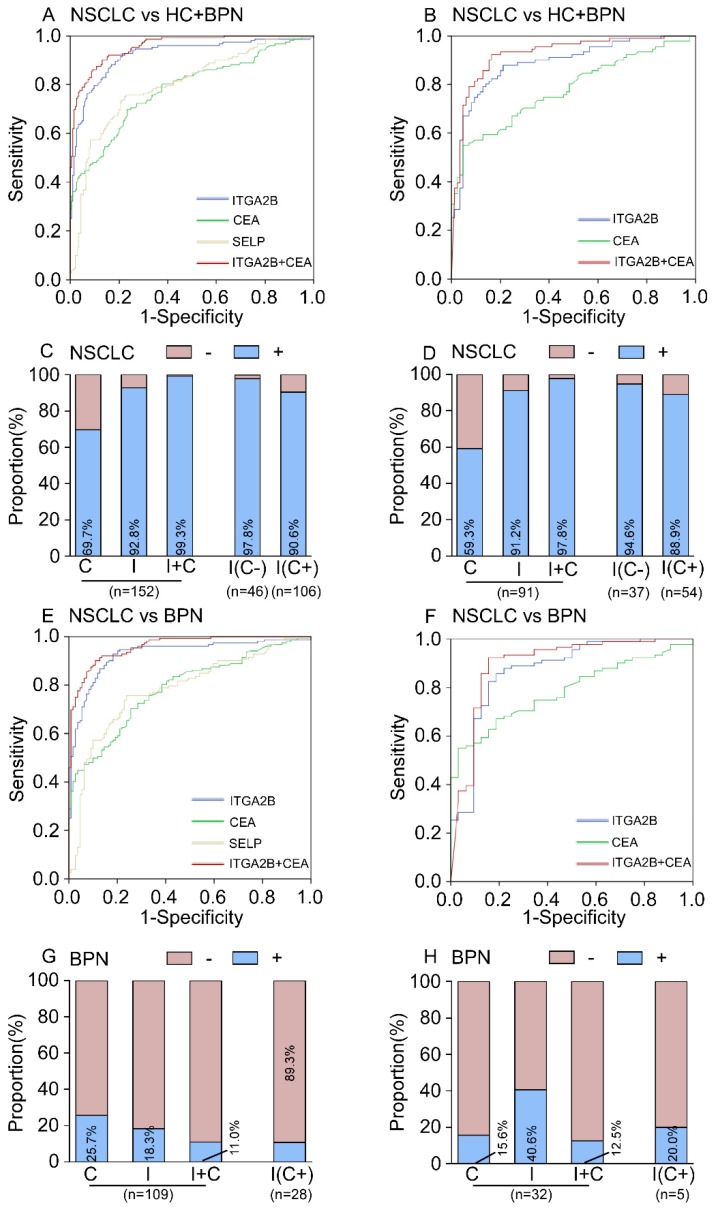
** Diagnostic outcomes for platelet ITGA2B, SELP mRNA and serum CEA in the NSCLC.** (A) ROC curve for platelet ITGA2B, SELP mRNA, serum CEA or combination of ITGA2B and CEA with NSCLC versus all controls in the test cohort. (B) ROC curve for platelet ITGA2B, SELP mRNA, serum CEA or combination of ITGA2B and CEA with NSCLC versus all controls in the validation cohort. (C) The rate of positive results for CEA, ITGA2B, or both, in all patients with NSCLC, and for ITGA2B by CEA status in the test cohort. (D) The rate of positive results for CEA, ITGA2B, or both, in all patients with NSCLC, and for ITGA2B by CEA status in the validation cohort. (E) ROC curve for platelet ITGA2B, SELP mRNA, serum CEA or combination of ITGA2B and CEA with NSCLC versus BPN in the test cohort. (F) ROC curve for platelet ITGA2B, SELP mRNA, serum CEA or combination of ITGA2B and CEA with NSCLC versus BPN in the validation cohort. (G) The rate of positive results for CEA and ITGA2B for patients with BPN, and for ITGA2B by CEA status in the test cohort. (H) The rate of positive results for CEA and ITGA2B for patients with BPN, and for ITGA2B by CEA status in the validation cohort. ROC: receiver operating characteristics; NSCLC: non-small cell lung cancer; BPN: benign pulmonary nodules; HC: healthy controls; C: CEA, carcinoembryonic antigen; I: ITGA2B, Integrin, Alpha 2b; SELP: P-selectin.

ROC curves analyses illustrated that the levels of platelet ITGA2B mRNA were robust in discriminating the subjects with or without NSCLC, with an AUC value of 0.922 (95% CI, 0.892-0.952, Figure 4A). When the cutoff value was set to the optimal point (0.001759), the sensitivity and specificity was 92.8% and 78.6% (Table 2), respectively. For platelet SELP mRNA, the AUC value was 0.799 (95% CI, 0.752-0.847, Figure 4A), and the optimum cutoff value was 0.003346, with 75.7% sensitivity and 77.2% specificity (Table 2). In contrast, platelet-related parameters such as platelet counts (PLT), mean platelet volume (MPV) and immature platelet fraction (IPF) showed poor effect in distinguishing between NSCLC and controls (See in the supplement materials and Figure S5).

The AUC for the CEA was 0.785 (95% CI, 0.736-0.834, Figure 4A), which was notably smaller than that for platelet ITGA2B mRNA (*p* < 0.001), implying that platelet ITGA2B exceeded CEA and SELP in distinguishing the patients with NSCLC from controls. Using the 2.865 ng/mL cutoff value, CEA generated a sensitivity of 69.7% and a specificity of 77.2%. Predictive values and accuracy of the platelet ITGA2B, SELP mRNA and CEA for detecting NSCLC were shown in Table 2, which showed that the accuracy of platelet ITGA2B mRNA was the highest one. We further explored whether ITGA2B can supplement CEA, the most common clinical used tumor marker in NSCLC and routine physical.

Larger proportion of patients with NSCLC in the test cohort were positive for platelet ITGA2B than for CEA (141 [92.8%] vs 106 [69.7%] of 152 patients; Figure 4C). 46/152 of NSCLC patients were CEA-negative and the proportion increased significantly at the early stage (30.3% vs 44.6%, *p* = 0.003, See in the supplement materials). Among them, 45 (97.8%) of 46 CEA-negative patients with NSCLC had positive ITGA2B results (Figure 4C). The rate was 96 of 106 (90.6%) in CEA-positive patients (Figure 4C). The ROC curves for ITGA2B indicated a higher diagnostic effect of NSCLC in CEA-negative patients than CEA-positive patients (CEA^-^ vs all controls: AUC = 0.951, 0.907-0.996; CEA^+^ vs all controls: AUC = 0.909, 0.873-0.946. Table S3 and Figure S6). Combining platelet ITGA2B and serum CEA, 151/152 (99.3%) of patients with NSCLC were positive, which was superior to that of either individual marker (Figure 4C). A binary logistic regression analysis was applied to construct a diagnostic model: Logit (*p* = NSCLC) = -3.66+91.87 ITGA2B+ 0.548 CEA. The model combined CEA with platelet ITGA2B, improving diagnostic capability for NSCLC of an individual marker. ROC curves analysis displayed the AUC for this combination was 0.957 (95% CI, 0.939-0.975), was similar to the combination of ITGA2B, CEA and SELP (Figure S7), but dramatically larger than that for ITGA2B, CEA only (Figure 4A) or either combination with SELP (Table S4). Using the 0.29188 cutoff, the sensitivity was 90.1%, and the specificity achieved 86.9% (Table 2), higher than other combinations (Table S4).

In discriminating the benign from malignant lung nodules, the results were similar, TEP ITGA2B had greater AUC, sensitivity, and specificity values than serum CEA and all values climbed when ITGA2B and CEA were combined (Figure 4E, Table 2). Dramatically, the rate of patients with BPN who had positive CEA results (25.7%, Figure 4G) was higher than those ITGA2B-positive (18.3%), and in CEA positive benign patients the proportions of negative results for platelet ITGA2B were extremely high (89.3%).

**Figure 5:**
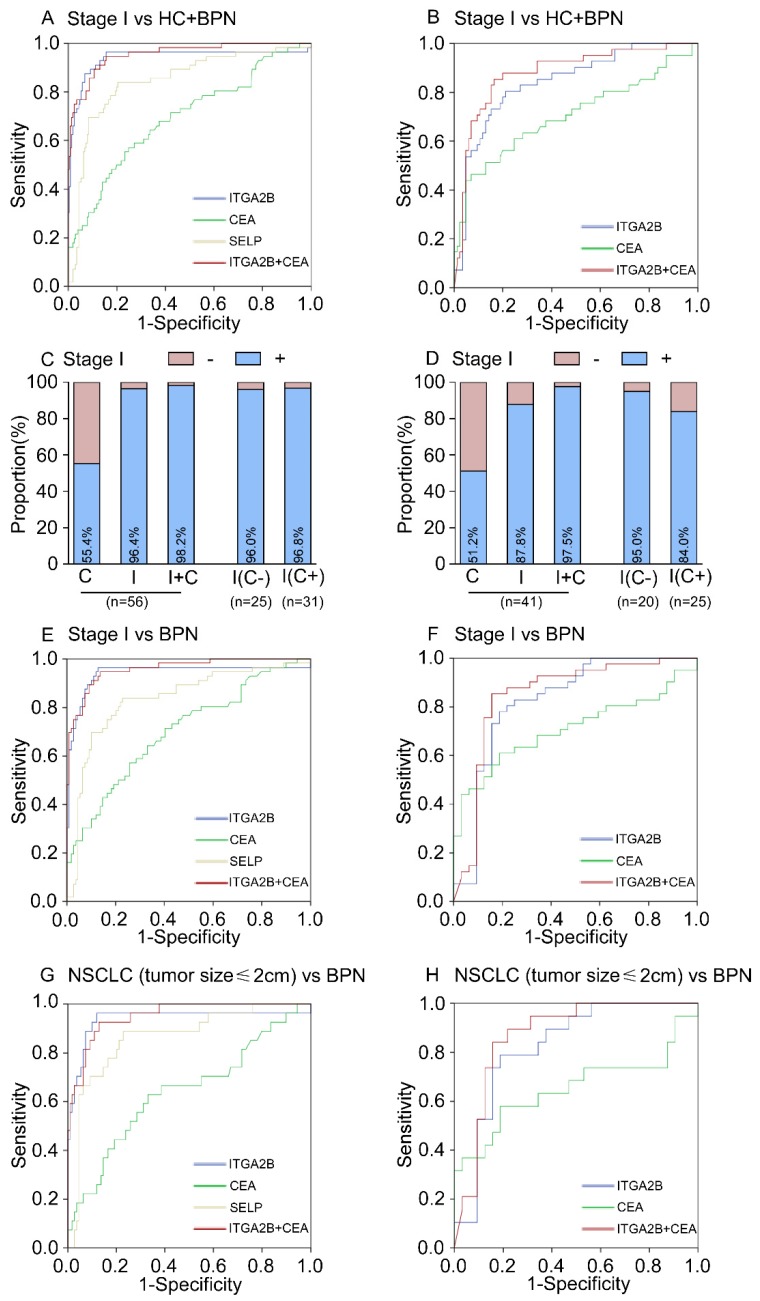
** Diagnostic outcomes for platelet ITGA2B, SELP mRNA and serum CEA in the stage I NSCLC.** (A) ROC curve for platelet ITGA2B, SELP mRNA, serum CEA or combination of ITGA2B and CEA with stage I NSCLC versus all controls in the test cohort. (B) ROC curve for platelet ITGA2B, SELP mRNA, serum CEA or combination of ITGA2B and CEA with stage I NSCLC versus all controls in the validation cohort. (C) The rate of positive results for CEA, ITGA2B, or both, in all patients with stage I NSCLC, and for ITGA2B by CEA status in the test cohort. (D) The rate of positive results for CEA, ITGA2B, or both, in all patients with stage I NSCLC, and for ITGA2B by CEA status in the validation cohort. (E) ROC curve for platelet ITGA2B, SELP mRNA, serum CEA or combination of ITGA2B and CEA with stage I NSCLC versus BPN in the test cohort. (F) ROC curve for platelet ITGA2B, SELP mRNA, serum CEA or combination of ITGA2B and CEA with stage I NSCLC versus BPN in the validation cohort. (G) ROC curve for platelet ITGA2B, SELP mRNA, serum CEA or combination of ITGA2B and CEA with stage I NSCLC with a solitary small tumour (≤ 2cm) versus BPN in the test cohort. (H) ROC curve for platelet ITGA2B, SELP mRNA, serum CEA or combination of ITGA2B and CEA with stage I NSCLC with a solitary small tumour (≤ 2cm) versus BPN in the validation cohort. ROC: receiver operating characteristics; NSCLC: non-small cell lung cancer; BPN: benign pulmonary nodules; HC: healthy controls; C: CEA, carcinoembryonic antigen; I: ITGA2B, Integrin, Alpha 2b; SELP: P-selectin.

In the test cohort, 56 (36.8%) of 152 patients with NSCLC were stage I. The relative expression levels of ITGA2B and SELP mRNA remained improved when only stage I lung cancer detection was involved (*p* < 0.001, Figure 3). ITGA2B enhanced differential diagnosis of stage I NSCLC from all controls and from controls with benign pulmonary nodules, compared with SELP or CEA (Figure 5, Table 2, Stage I vs all controls: ITGA2B, AUC = 0.940; SELP, AUC = 0.846; CEA, AUC = 0.694; Stage I vs BPN: ITGA2B, AUC = 0.941; SELP, AUC = 0.833; CEA, AUC = 0.702). The sensitivity, specificity and predictive values for TEP ITGA2B were also higher than those for SELP or CEA (Table 2). Moreover, a substantially higher rate of patients with stage I NSCLC had positive results for TEP ITGA2B than for serum CEA (ITGA2B, 96.4%, 54/56; CEA, 55.4%, 31/56). Among CEA-negative patients with stage I NSCLC, 24 of 25 (96.0%) had positive platelet ITGA2B results, as did 30 of 31(96.8%) CEA positive patients. The ROC curves for platelet ITGA2B indicated a diagnosis of stage I lung cancer irrespective of CEA status (supplement materials, Table S3 and Figure S8). Test both platelet ITGA2B and CEA improved the diagnostic capability for NSCLC than individual marker, with an AUC of 0.956 (stage I vs all control), or 0.958 (stage I vs benign).

In current clinics, it is very difficult to distinguish NSCLC patients with only a single lesion less than 2cm from benign pulmonary nodules. We further evaluated the performance of platelet ITGA2B in discriminating solitary pulmonary nodule (SPN). In 27 patients with malignant pulmonary nodules who had only one tumour less than 2 cm, the AUC for platelet ITGA2B was 0.937 (95% CI, 0.865-1.000) with sensitivity of 96.3% and specificity of 81.7%, compared with benign pulmonary nodules patients (Figure 5G, Table 2), was larger than that for CEA (0.640, 0.515-0.765), their combination yielded a rather higher AUC, 0.951 (0.913-0.989) in discriminating SPN.

When the same threshold applied to the blinded validation cohort, we observed similar results to those in the test cohort. Platelet ITGA2B had pinpoint diagnostic accuracy for all NSCLC, stage I NSCLC, and NSCLC with only one tumour of 2 cm or less (Table 2, Figures 4, 5). In addition, the validation cohort verified the ability of ITGA2B to diagnose NSCLC in CEA-negative patients, including those with stage I disease (Table S3 and Figure S6, 8).

The ability of ITGA2B to discriminate patients with NSCLC from those with benign pulmonary nodule(s), especially in the discrimination of small solitary pulmonary nodule was also affirmed (Figure 5H). The improvement in diagnostic precision for NSCLC by assessment of platelet ITGA2B and CEA together was confirmed in the validation cohort as well (Figures 4, 5).

ROC analysis also showed that testing of SELP achieved a good diagnostic accuracy for NSCLC and other NSCLC subgroups in both cohorts (Figure 4, 5, Table 2 and Figure S9).

**Figure 6:**
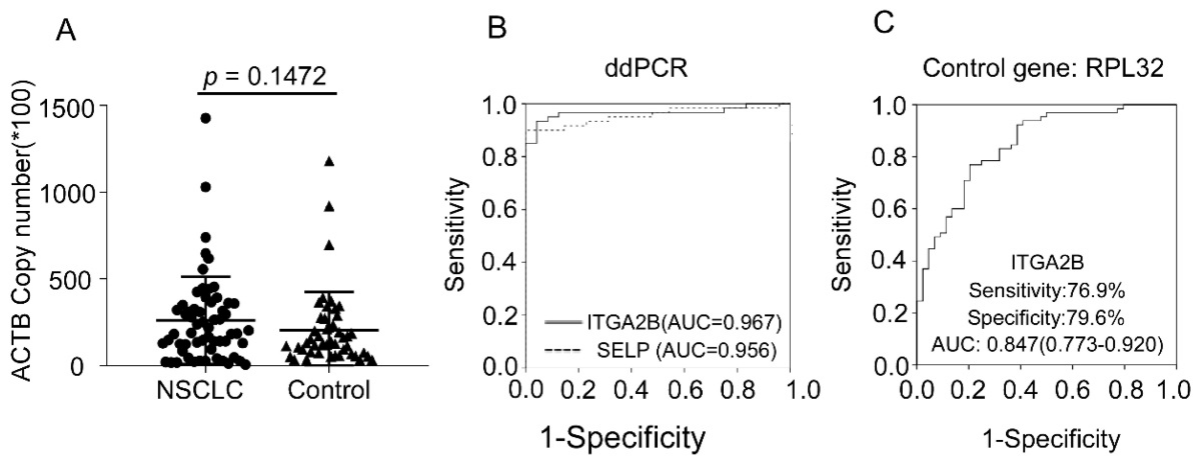
** Diagnostic outcomes for platelet ITGA2B, and SELP mRNA in the NSCLC using ddPCR and RPL32 reference gene.** (A) The absolute copy number of β-actin derived via ddPCR. (B) ROC curve for platelet ITGA2B, and SELP mRNA with NSCLC versus all controls in the subgroup of test cohort using ddPCR. (C) ROC curve for platelet ITGA2B, and SELP mRNA with NSCLC versus all controls in the subgroup of test cohort using RPL32 reference gene. ROC: receiver operating characteristics; NSCLC: non-small cell lung cancer; ddPCR: Droplet digital PCR; AUC: areas under the curves; ITGA2B: Integrin, Alpha 2b; SELP: P-selectin.

We verified the results through more precise methodology, ddPCR, an approach that allows absolute quantification. With this more precise and sensitive approach, we observed similar results. The copy number of β-actin between NSCLC and controls was similar, confirming that β-actin could be used as the internal control gene. The ability of ITGA2B and SELP to distinguish patients with NSCLC from control was confirmed, yielded an AUC of 0.967 and 0.956, respectively (Figure 6). Spearman correlation revealed that the results derived from two methods were significantly associated (*p* < 0.001).

RPL32 was also reported as an internal control gene in platelet study^34^. We additionally assessed RPL32 in 65 NSCLC, 28 BPN and 16 controls from test cohort. The results calculated were related with the data derived when actin was the internal gene (*p* < 0.001) and confirmed the ability of ITGA2B to diagnose NSCLC patients with an AUC of 0.847(0.773-0.920), sensitivity of 76.9% and specificity of 79.5% (Figure 6).

### Association of platelet ITGA2B, SELP with clinicopathologic charateristics and survival in patients with NSCLC

While the optimal cutoff value (0.001749) of platelet-ITGA2B mRNA was used to categorize NSCLC patients into high-level or low-level group, χ^2^ tests showed that there were no differences in clinicopathologic parameters including histological type between patients with NSCLC and platelet-ITGA2B mRNA overall (all at *p* > 0.05 except smoking status in validation cohort, Table S5 and Figure S10). No significant association of SELP with clinicopathologic parameters was observed (Table S6 and Figure S10).

Two-hundred and forty-three patients with NSCLC, the overall enrolled in our study were prospectively followed up with the mean duration of 26.82 (range 0.58-36) months, and the cumulative 3-year OS rate was 76.3%.

**Figure 7:**
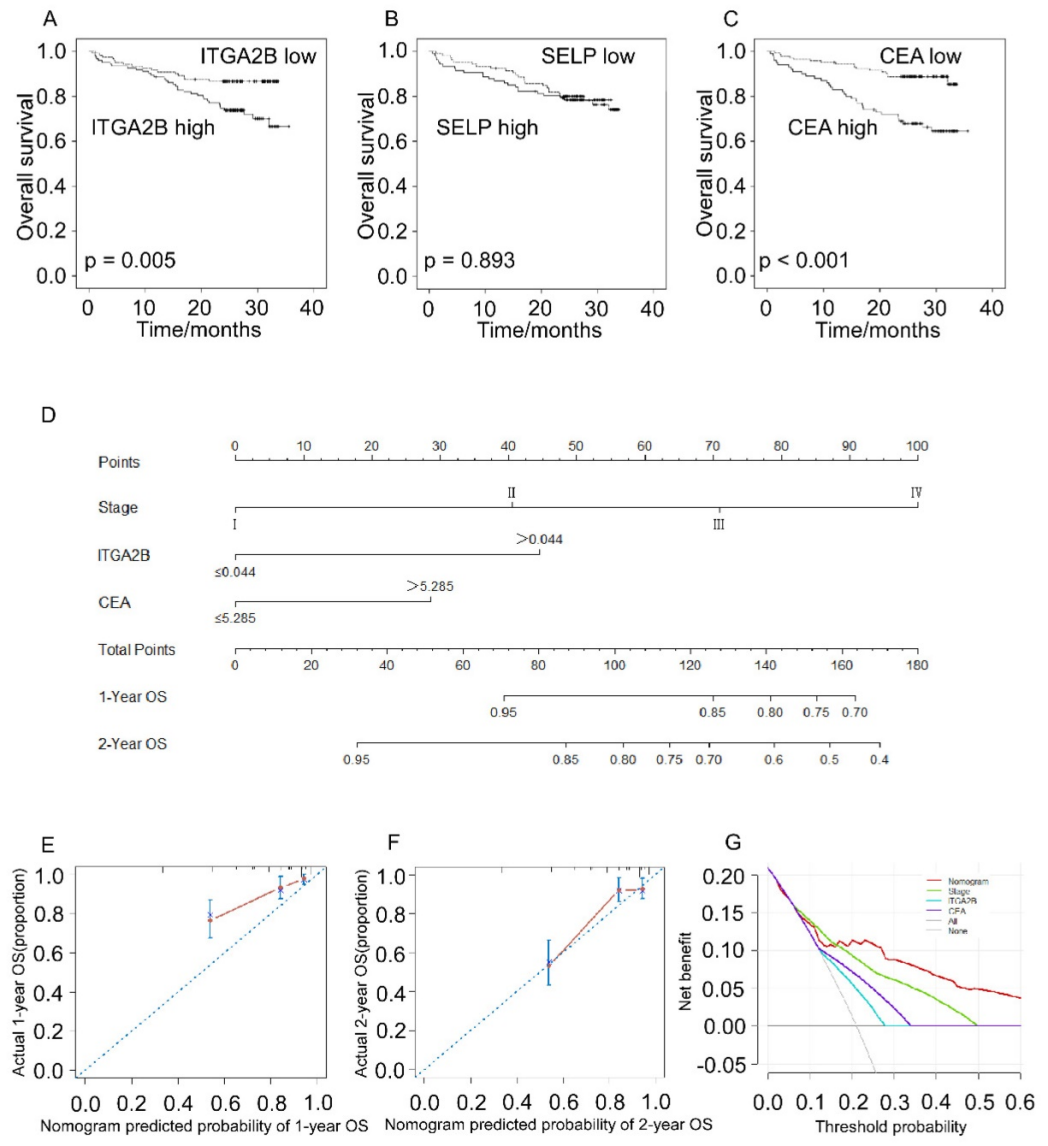
** Prognostic prediction for OS according to platelets ITGA2B, platelets SELP, CEA and nomogram.** Kaplan-Meier curves for OS according to platelets ITGA2B (A), platelets SELP (B) and CEA (C); (D) Nomogram model predicting 1-, and 2- year OS in NSCLC patients; The calibration curves for predicting patient OS at one year (E), and two years (F); (G) Decision curve analysis for 2-year survival predictions. OS: overall survival; ITGA2B: Integrin, Alpha 2b; SELP: P-selectin; CEA: carcinoembryonic antigen

Patients with NSCLC with higher platelet ITGA2B mRNA expression levels had remarkably worse OS rate compared to patients with lower platelet ITGA2B (71.5% vs. 86.7%; *p* = 0.005), and CEA showed the similar result (88.1% vs. 66.0%; *p* < 0.001) whereas SELP not (77.4% vs 78.5%, *p* = 0.893), seen in Figure 7. We next employed univariable Cox proportional hazard regression model (Table 3) and the results showed that OS was remarkably associated with platelet ITGA2B mRNA (*p* = 0.006), clinical stage (*p* < 0.001), and CEA (*p* < 0.001). Strikingly, multivariable Cox analysis illustrated that only platelet ITGA2B mRNA (HR, 2.295, 95% CI, 1.263-4.170, *p* = 0.006), and clinical stage (HR, 1.973, 95% CI, 1.500-2.595, *p* < 0.001) retained independent prognostic value.

The nomogram model, which integrated stage, ITGA2B, and CEA, showed commendable accuracy for predicting OS proportion of NSCLC, with a C-index of 0.756 (Figure 7D, 95% CI, 0.686-0.827). The calibration plots revealed good prediction of 1- and 2-year OS (Figure 7E, F). Decision curve analysis (Figure 7G) displayed that the use of a nomogram which took account of stage, CEA and ITGA2B had a higher net clinical benefit. And it also across a wider range of threshold probabilities to predict OS compared to stage, CEA and ITGA2B.

## Discussion

We have shown that measurement of platelet derived ITGA2B mRNA has diagnostic value for NSCLC better than that of CEA, especially for patients with CEA-negative status and stage I NSCLC. Platelet derived SELP mRNA also shown its diagnostic value in NSCLC, which was relevant statistically and inferior to ITGA2B in the estimate of NSCLC presence. Although CEA performed better than CYFRA21-1, there were still more than 30% of all patients with NSCLC were CEA negative, consistent with other studies^11^. Diagnosis and assessment of treatment response are still difficult with current methods. Thereby, combined measurement of platelet ITGA2B mRNA and serum CEA levels could enhance results.

Results were negative for platelet ITGA2B in most CEA-positive BPN participants, thereby, patients with these non-malignant diseases could be discriminated from those with NSCLC. The expected frequency of lung nodule detection (20% to 60% of high-risk individuals), and the fact that the large majority of these lung nodules are benign (up to 96%), make the management of such indeterminate pulmonary nodules challenging. And the value of CEA in early identification of pulmonary nodule was limited. Therefore, measurement of TEP ITGA2B mRNA was meritorious to help clinicians make a differential diagnosis of NSCLC in these high-risk populations. We also evaluated its effect in distinguishing small solitary pulmonary nodule, the subgroup of BPN with more difficult challenge to be identified in CT. Many benign lesions can also manifest as SPN such as pulmonary TB, pneumonia, pulmonary abscess and inflammatory pseudotumour 35. Although the population of less than 2cm SPN were of small sample size, we still could recognize the trend that the combination of ITGA2B and CEA was useful for differential diagnoses from NSCLC.

We verified the results in another independent population and through different methodology. The similar results to those in the test cohort were observed, the ability of ITGA2B and SELP to distinguish patients with NSCLC from those with non-malignant pulmonary nodules and healthy controls were confirmed. The different technique we took advantage for validation was the droplet digital PCR (ddPCR), an approach that allows absolute quantification without the need for internal/external normalization^36^. DdPCR is not affected by PCR inhibitors and extremely sensitive and precise. In our study, we controlled several measures in the two PCR assay, including RNA extraction, cDNA synthesis, primer design and reference gene selection and so on^37^, then compared the two results derived from respective method and found they were highly relevant. As the levels of the two platelet-derived mRNA were rather high, and qRT-PCR still represented most frequently used quantification platform, we recommended the qRT-PCR as survey methodology in the following research. This simpler, cheaper test is easier to be carried out for screening. Besides, we selected another reference gene, RPL32^34^, ^38^ and verify the results as well.

As a result, ITGA2B and SELP mRNA were not related to stage. Consequently, the diagnostic accuracy of them was similar in the stage I NSCLC and all NSCLC patients. Interestingly, although they took part in platelet activation, the isolated platelet pellets showed no activation, as measured by FC analysis and WB, indicating that TEP mRNA, but not protein served as source of liquid biopsy. The tumor platelets could perhaps behave in a 'semi-activational state'^23^ but absence of activation, and overexpressed the ITGA2B and SELP mRNA but not protein in the initial of tumorgenesis. However, this assumption needs to be tested further. We believe, though, that TEP ITGA2B and SELP have great potential to be a diagnostic mRNA biomarker for NSCLC because its accuracy in the test cohort was confirmed in another independent cohort, even with a more precise method.

For the first time, prognostic analyses demonstrated that a high TEP ITGA2B was an independent risk factor of a poorer prognosis with respect to OS in patients with NSCLC. Moreover, a nomogram model integrating ITGA2B, CEA and clinical stage was successfully established for predicting OS in NSCLC patients and showed improved prognostic accuracy. Continued follow-up of the participants will be performed in the future study.

Platelets have long been considered as a potential diagnostic tool in cancer. Previous studies have illustrated that the platelet count, platelet size and platelet subpopulations can already provide clinically relevant information about the presence of cancer^39^. However, we did not observe any significant differences in platelet counts, platelet size, and younger platelets between healthy controls and patients in our population, which may due to the heterogeneity of the study populations. Tumor cells can, directly and indirectly, impose changes on platelet RNA and protein content. TEP RNA, emerging as a new means of liquid biopsy, was borne out to reflect the presence of cancer and thus give hope to many that they might precede protein markers as a cancer-specific biomarker^22^, ^23^. They were reported to distinguish cancer patients from healthy controls with 96% accuracy. In addition, a swarm-intelligence, that is a TEP-RNA panel containing 698 RNAs were shown to be associated with the presence of NSCLC. The results were meritorious, however, this approach is cumulative SVM algorithms, involved in a substantial number of genes, that may limit its application due to its complication. Our study suggested a small quantity of TEP with high accuracy in NSCLC detection and moreover, differential and early diagnosis.

Either of platelet ITGA2B and SELP mRNA participated in almost all the top 20 enriched GO biological process in our dataset, implying that they had a profound effect with a great potential to be implemented in clinic application. ITGA2B, is a transmembrane glycoprotein that is expressed by megakaryocytes and platelets^40^. It is required for platelet aggregation, and defects lead to thrombasthenia. Except for adhesion, integrins are also known to participate in cell-surface mediated signalling. It has been reported to be pivotal for hematogenous metastasis of human breast carcinoma MDA-MB-231 cells^41^ and predictive of prognosis in clear cell renal cell carcinoma patients^42^. SELP, is found constitutively in a pre-formed state in the alpha granules of platelets and on the surfaces of activated endothelial platelets^43^. Tumor cells interact with platelets mainly via the platelet activation receptor P-selectin. A number of studies have reported that levels of the expression of P-selectin on platelets and sP-Selectin in biological fluids may be elevated in subjects with a variety of cancers, including renal carcinoma and colorectal carcinoma^44^. These two TEP, especially ITGA2B implied immense potential in clinical application, and our study demonstrated they could serve as great NSCLC diagnostic marker. However, at present, the underlying mechanisms of their origin, or TEP source are not fully understood. The most common hypothesis is that: platelets actively maintain their RNA content while in circulation, including via the use of platelet RNA splicing^45^, circularization^46^, and decay^47^, possibly in response to external queues.

In our study, we isolated platelet as previously advised by others 22, 23. Platelet isolation was executed with simple step and the whole blood could be stored up to 48 h at room temperature while still maintaining high-quality RNA and the dominant RNA signatures, which revealed its feasibility in clinical applications.

For the first time, we reported the clinically diagnostic and prognostic relevance of ITGA2B as a platelet RNA marker for NSCLC in a test cohort and an independent validation cohort. Although our results are promising, there are also some limitations in our study. Larger samples are required to rule out the incidence of NSCLC bias, the type of control bias, and selection bias in the following research. And forward investigations should elucidate the biological role and oncological significance of TEP ITGA2B more clearly.

In conclusion, the current study clearly reveals that the platelet derived mRNA, ITGA2B and SELP are significantly increased in primary lung cancer patients thus could discriminating NSCLC and controls, and the combination of ITGA2B and CEA even performs better. This new means of liquid biopsy provides novel insight for NSCLC and useful information for clinicians.

## Conclusions

TEP ITGA2B combined with CEA could be useful in NSCLC detection and prognosis prediction.

## Supplementary Material

Supplementary figures and tables.

## Figures and Tables

**Table 1:** The clinicopathologic characteristics of NSCLC patients

Characteristics	Test(n=152)		Validation (n=91)		*p* value
	n	%	n	%	
Age, years					0.679
Median	58.5		60		
IQR	52-65		53-65		
Sex					0.825
Male	88	58	54	59	
Female	64	42	37	41	
pT classification					0.339
pT1	63	41	32	35	
pT2-pT4	83	55	55	60	
Missing	6	4	4	4	
pN classification					0.242
pN0	71	47	49	54	
pN1-pN3	72	47	36	40	
Missing	9	6	6	7	
Metastasis					0.513
pM0	119	78	72	79	
pM1	33	22	16	18	
Missing			3	3	
Clinical stage					0.206
Stage I	56	37	41	45	
Stage II-Stage IV	96	63	50	55	
Histology type					**0.049**
ADC	137	90	74	81	
SqCC	15	10	17	19	
Smoke status					0.358
Yes	71	47	37	41	
No	81	53	54	59	
PLT					0.87
PLT ≥ 300	37	24	23	25	
PLT < 300	115	76	68	75	

NSCLC: non-small cell lung cancer; ITGA2B: Integrin, Alpha 2b; CEA: carcinoembryonic antigen; ADC: adenocarcinoma; SqCC: squamous cell carcinoma

**Table 2:** Results for measurement of platelets ITGA2B, SELP mRNA, serum CEA or combination of ITGA2B and CEA in the diagnosis of NSCLC

	Test								Validation						
	AUC(95%CI)	SN(%)	SP(%)	PPV(%)	NPV(%)	Positive LR	Negative LR		AUC(95%CI)	SN(%)	SP(%)	PPV(%)	NPV(%)	Positive LR	Negative LR
**NSCLC vs BPN and HC**															
ITGA2B	0.922(0.892-0.952)	92.8	78.6	76.2	93.6	4.34	0.09		0.888(0.838-0.938)	91.2	56.5	69.2	85.7	2.10	0.16
SELP	0.799(0.752-0.847)	75.7	77.2	71.0	81.1	3.32	0.31		0.716(0.616-0.815)	96.7	43.1	67.9	91.7	1.70	0.08
CEA	0.783(0.733-0.832)	69.7	76.7	40.2	50.0	2.99	0.40		0.779(0.711-0.847)	59.3	81.2	77.1	65.1	3.15	0.50
ITGA2B+CEA	0.957(0.939-0.975)	90.1	86.9	83.5	92.3	6.88	0.11		0.925(0.884-0.965)	85.7	87.1	87.6	85.1	6.64	0.16
**NSCLC vs BPN**															
ITGA2B	0.927(0.893-0.960)	92.8	81.7	87.6	89.0	5.07	0.09		0.871(0.792-0.949)	91.2	59.4	86.5	70.4	2.25	0.15
SELP	0.782(0.724-0.839)	75.7	76.1	81.6	69.2	3.17	0.32		0.825(0.722-0.928)	96.7	43.8	86.8	77.8	1.72	0.08
CEA	0.786(0.732-0.840)	69.7	74.3	79.1	63.8	2.71	0.41		0.785(0.706-0.865)	59.3	84.4	91.5	42.2	3.80	0.48
ITGA2B+CEA	0.959(0.938-0.979)	90.1	89.0	91.9	86.6	8.19	0.11		0.898(0.823-0.972)	85.7	87.5	95.1	68.3	6.86	0.16
**Stage I NSCLC vs BPN and HC**															
ITGA2B	0.940(0.890-0.989)	96.4	78.6	55.1	98.8	4.50	0.05		0.842(0.767-0.916)	87.8	56.5	49.3	90.6	2.02	0.22
SELP	0.846(0.784-0.908)	83.9	77.2	50.0	94.6	3.68	0.21		0.642(0.520-0.763)	93.1	43.1	48.2	91.7	1.64	0.16
CEA	0.694(0.611-0.776)	55.4	76.7	39.2	86.3	2.38	0.58		0.704(0.597-0.812)	51.2	81.2	56.8	77.5	2.72	0.60
ITGA2B+CEA	0.955(0.925-0.984)	91.1	86.9	65.4	97.3	6.95	0.10		0.879(0.812-0.947)	75.6	87.1	73.8	88.1	5.86	0.28
**Stage I NSCLC vs BPN**															
ITGA2B	0.940(0.890-0.991)	96.4	81.7	73.0	97.8	5.27	0.04		0.825(0.721-0.930)	87.8	59.4	73.5	79.2	2.16	0.21
SELP	0.833(0.763-0.904)	83.9	76.1	64.4	90.2	3.51	0.21		0.750(0.604-0.896)	93.1	43.8	75.0	77.8	1.66	0.16
CEA	0.702(0.617-0.788)	55.4	74.3	52.5	76.4	2.16	0.60		0.708(0.588-0.828)	51.2	84.4	80.8	57.4	3.28	0.58
ITGA2B+CEA	0.958(0.928-0.988)	91.1	89.0	81.0	95.1	8.28	0.10		0.848(0.747-0.950)	75.6	87.5	88.6	73.7	6.05	0.28
**≤2cm NSCLC vs BPN**															
ITGA2B	0.937(0.865-1.000)	96.3	81.7	56.5	98.9	5.26	0.05		0.826(0.711-0.940)	89.5	59.4	56.7	90.5	2.20	0.18
SELP	0.862(0.782-0.942)	88.9	76.1	48.0	96.5	3.72	0.15		0.798(0.637-0.959)	100.0	43.8	59.1	100.0	1.78	0.00
CEA	0.640(0.515-0.765)	48.1	74.3	31.7	85.3	1.87	0.70		0.653(0.476-0.830)	42.1	84.4	61.5	71.1	2.70	0.69
ITGA2B+CEA	0.951(0.913-0.989)	88.9	89.0	66.7	97.0	8.08	0.12		0.869(0.769-0.970)	73.7	87.5	77.8	84.8	5.90	0.30

AUC: area under curve; SN:sensitivity; SP: specificity; PPV: positive predictive value; NPV: negative predictive value; NSCLC: non-small cell lung cancer; BPN: benign pulmonary nodules; HC: healthy controls; ITGA2B: Integrin, Alpha 2b; SELP: P-selectin; CEA: carcinoembryonic antigen. *The diagnostic cutoff values of platelets ITGA2B, SELP mRNA, serum CEA or combination of ITGA2B and CEA were 0.001759,0.003346,2.865ng/ml and 0.29188, respectively.

**Table 3:** Univariate and multivariate analysis of overall survival

Variables	Univariate Analysis				Multivariate Analysis		
	HR	CI	*p*		HR	CI	*p*
Age	1.028	1.000-1.057	0.05				
Sex	0.675	0.377-1.209	0.187				
Smoking status	0.74	0.427-1.281	0.282				
Histology type	1.26	0.592-2.682	0.180				
Clinical stage	2.128	1.638-2.763	**< 0.001**		1.973	1.500-2.595	**< 0.001**
ITGA2B	2.3	1.272-4.159	**0.006**		2.295	1.263-4.170	**0.006**
CEA	3.226	1.802-5.777	**< 0.001**		1.813	0.976-3.369	0.060

Variables: Age, ≥ 60 vs < 60; Sex, female vs male; Smoking status, smoker vs non-smoker; Histology type, Adenocarcinoma vs Non-Adenocarcinoma; stage, Ⅳ vs Ⅲ vs Ⅱ vs I, ITGA2B: Integrin, Alpha 2b; CEA: carcinoembryonic antigen.

## Abbreviations

ITGA2B: Integrin Alpha 2b
NSCLC: non-small-cell lung cancer
TEPs: tumor-educated blood platelets
PCR: Polymerase Chain Reaction
AUC: area under the curve
RPL32: Ribosomal Protein L32
CEA: carcinoembryonic antigen
ADC: adenocarcinoma
SqCC: squamous cell carcinoma
LDCT: low-dose computed tomography
IPN: indeterminate pulmonary nodule
CYFRA21-1: cytokeratin-19 fragment
SVM: support vector machine
SYSUCC: Sun Yat-Sen University Cancer Center
BPN: benign pulmonary nodules
HC: healthy control
CT: computed tomography
PRP: platelet-rich plasma
DC: direct current
ECLIA: electrochemiluminescence immunoassay
GO: Gene Ontology
KEGG: Kyoto Encyclopedia of Genes and Genomes
Ct: threshold cycle
CV: coefficient of variation
ROC: Receiver operating characteristics
HR: hazard ratio
RPKM: Reads Per Kilo bases per Million reads
K-M analyses: Kaplan-Meier analyses
MPV: mean platelet volume
IPF: immature platelet fraction
SPN: solitary pulmonary nodule

## References

[1] Siegel RL, Miller KD, Jemal A (2018). Cancer statistics, 2018. CA Cancer J Clin.

[2] Chen W, Zheng R, Baade PD, Zhang S, Zeng H, Bray F (2016). Cancer statistics in China, 2015. CA Cancer J Clin.

[3] Lai Y, Wang X, Zeng T, Xing S, Dai S, Wang J (2018). Serum VEGF levels in the early diagnosis and severity assessment of non-small cell lung cancer. J Cancer.

[4] Tammemagi MC, Lam S (2014). Screening for lung cancer using low dose computed tomography. BMJ.

[5] National Lung Screening Trial Research T, Aberle DR, Adams AM, Berg CD, Black WC, Clapp JD (2011). Reduced lung-cancer mortality with low-dose computed tomographic screening. N Engl J Med.

[6] Patz EF Jr, Pinsky P, Gatsonis C, Sicks JD, Kramer BS, Tammemagi MC (2014). Overdiagnosis in low-dose computed tomography screening for lung cancer. JAMA Intern Med.

[7] Wiener RS, Schwartz LM, Woloshin S, Welch HG (2011). Population-based risk for complications after transthoracic needle lung biopsy of a pulmonary nodule: an analysis of discharge records. Ann Intern Med.

[8] Sozzi G, Boeri M, Rossi M, Verri C, Suatoni P, Bravi F (2014). Clinical utility of a plasma-based miRNA signature classifier within computed tomography lung cancer screening: a correlative MILD trial study. J Clin Oncol.

[9] Xie YJ, Zhang Y, Du LT, Jiang XM, Yan SZ, Duan WL (2018). Circulating long noncoding RNA act as potential novel biomarkers for diagnosis and prognosis of non-small cell lung cancer. Mol Oncol.

[10] Ren SX, Zhang SC, Jiang T, He YY, Ma ZY, Cai HR (2018). Early detection of lung cancer by using an autoantibody panel in Chinese population.

[11] Molina R, Marrades RM, Auge JM, Escudero JM, Vinolas N, Reguart N (2016). Assessment of a Combined Panel of Six Serum Tumor Markers for Lung Cancer. Am J Resp Crit Care.

[12] Zamcheck N, Pusztaszeri G (1975). CEA, AFP and other potential tumor markers. CA Cancer J Clin.

[13] Ludwig JA, Weinstein JN (2005). Biomarkers in cancer staging, prognosis and treatment selection. Nat Rev Cancer.

[14] Sajid KM, Parveen R, Durr e S, Chaouachi K, Naeem A, Mahmood R (2007). Carcinoembryonic antigen (CEA) levels in hookah smokers, cigarette smokers and non-smokers. J Pak Med Assoc.

[15] Correa-Gallego C, Warshaw AL, Fernandez-del Castillo C (2009). Fluid CEA in IPMNs: A useful test or the flip of a coin?. Am J Gastroenterol.

[16] Sreenarasimhaiah J, Lara LF, Jazrawi SF, Barnett CC, Tang SJ (2009). A comparative analysis of pancreas cyst fluid CEA and histology with DNA mutational analysis in the detection of mucin producing or malignant cysts. JOP.

[17] Li X, Asmitananda T, Gao L, Gai D, Song Z, Zhang Y (2012). Biomarkers in the lung cancer diagnosis: a clinical perspective. Neoplasma.

[18] Plebani M, Basso D, Navaglia F, De Paoli M, Tommasini A, Cipriani A (1995). Clinical evaluation of seven tumour markers in lung cancer diagnosis: can any combination improve the results?. Br J Cancer.

[19] Xu XR, Zhang D, Oswald BE, Carrim N, Wang X, Hou Y (2016). Platelets are versatile cells: New discoveries in hemostasis, thrombosis, immune responses, tumor metastasis and beyond. Crit Rev Clin Lab Sci.

[20] McAllister SS, Weinberg RA (2014). The tumour-induced systemic environment as a critical regulator of cancer progression and metastasis. Nat Cell Biol.

[21] Calverley DC, Phang TL, Choudhury QG, Gao B, Oton AB, Weyant MJ (2010). Significant downregulation of platelet gene expression in metastatic lung cancer. Clin Transl Sci.

[22] Best MG, Sol N, Kooi I, Tannous J, Westerman BA, Rustenburg F (2015). RNA-Seq of Tumor-Educated Platelets Enables Blood-Based Pan-Cancer, Multiclass, and Molecular Pathway Cancer Diagnostics. Cancer Cell.

[23] Best MG, Sol N, 't Veld SGJGI, Vancura A, Muller M, Niemeijer ALN (2017). Swarm Intelligence-Enhanced Detection of Non-Small-Cell Lung Cancer Using Tumor-Educated Platelets. Cancer Cell.

[24] Liu WT, Xiang LP, Zheng TK, Jin JJ, Zhang G (2018). TranslatomeDB: a comprehensive database and cloud-based analysis platform for translatome sequencing data. Nucleic Acids Res.

[25] Xie C, Mao X, Huang J, Ding Y, Wu J, Dong S (2011). KOBAS 2.0: a web server for annotation and identification of enriched pathways and diseases. Nucleic Acids Res.

[26] Fei F, Li XF, Xu L, Li DY, Zhang ZP, Guo X (2014). CD147-CD98hc Complex Contributes to Poor Prognosis of Non-Small Cell Lung Cancer Patients Through Promoting Cell Proliferation Via the PI3K/Akt Signaling Pathway. Ann Surg Oncol.

[27] Ewing RM, Chu P, Elisma F, Li H, Taylor P, Climie S (2007). Large-scale mapping of human protein-protein interactions by mass spectrometry.

[28] Li HP, Wu HJ, Zhang HF, Li Y, Li S, Hou Q (2017). Identification of curcumin-inhibited extracellular matrix receptors in non-small cell lung cancer A549 cells by RNA sequencing.

[29] Qi C, Wei B, Zhou W, Yang Y, Li B, Guo S (2015). P-selectin-mediated platelet adhesion promotes tumor growth. Oncotarget.

[30] Goreczny GJ, Ouderkirk-Pecone JL, Olson EC, Krendel M, Turner CE (2017). Hic-5 remodeling of the stromal matrix promotes breast tumor progression. Oncogene.

[31] Wills B, Cardona AF, Rojas L, Ruiz-Patino A, Arrieta O, Reguart N (2017). Survival Outcomes According to TIMP1 and EGFR Expression in Heavily Treated Patients with Advanced Non-small Cell Lung Cancer who Received Biweekly Irinotecan Plus Bevacizumab. Anticancer Res.

[32] Sarhadi VK, Lahti L, Scheinin I, Ellonen P, Kettunen E, Serra M (2014). Copy Number Alterations and Neoplasia-Specific Mutations in MELK, PDCD1LG2, TLN1, and PAX5 at 9p in Different Neoplasias. Gene Chromosome Canc.

[33] Tang C, Wang J, Wei Q, Du YP, Qiu HP, Yang C (2018). Tropomyosin-1 promotes cancer cell apoptosis via the p53-mediated mitochondrial pathway in renal cell carcinoma. Oncol Lett.

[34] Wang XK, Hsu MY, Steinbacher TE, Monticello TM, Schumacher WA (2007). Quantification of platelet composition in experimental venous thrombosis by real-time polymerase chain reaction. Thromb Res.

[35] Lazarus DR, Ost DE (2013). The Solitary Pulmonary Nodule-Deciding When to Act?. Semin Resp Crit Care.

[36] Hindson CM, Chevillet JR, Briggs HA, Gallichotte EN, Ruf IK, Hindson BJ (2013). Absolute quantification by droplet digital PCR versus analog real-time PCR. Nat Methods.

[37] Tichopad A, Kitchen R, Riedmaier I, Becker C, Stahlberg A, Kubista M (2009). Design and Optimization of Reverse-Transcription Quantitative PCR Experiments. Clin Chem.

[38] Shen YM, Li Y, Ye F, Wang FF, Lu WG, Xie X (2010). Identification of suitable reference genes for measurement of gene expression in human cervical tissues. Anal Biochem.

[39] Stone RL, Nick AM, McNeish IA, Balkwill F, Han HD, Bottsford-Miller J (2012). Paraneoplastic Thrombocytosis in Ovarian Cancer. New Engl J Med.

[40] Heidenreich R, Eisman R, Surrey S, Delgrosso K, Bennett JS, Schwartz E (1990). Organization of the gene for platelet glycoprotein IIb. Biochemistry.

[41] Zhao F, Li L, Guan L, Yang H, Wu C, Liu Y (2014). Roles for GP IIb/IIIa and alphavbeta3 integrins in MDA-MB-231 cell invasion and shear flow-induced cancer cell mechanotransduction. Cancer Lett.

[42] Lu X, Wan F, Zhang H, Shi G, Ye D (2016). ITGA2B and ITGA8 are predictive of prognosis in clear cell renal cell carcinoma patients. Tumour Biol.

[43] Stenberg PE, McEver RP, Shuman MA, Jacques YV, Bainton DF (1985). A platelet alpha-granule membrane protein (GMP-140) is expressed on the plasma membrane after activation. J Cell Biol.

[44] Dymicka-Piekarska V, Matowicka-Karna J, Osada J, Kemona H, Butkiewicz AM (2006). Changes in platelet CD 62P expression and soluble P-selectin concentration in surgically treated colorectal carcinoma. Adv Med Sci.

[45] Denis MM, Tolley ND, Bunting M, Schwertz H, Jiang H, Lindemann S (2005). Escaping the nuclear confines: signal-dependent pre-mRNA splicing in anucleate platelets. Cell.

[46] Alhasan A, Izuogu OG, Al-Balool HH, Steyn JS, Evans A, Colzani M (2016). Circular RNA enrichment in platelets is a signature of transcriptome degradation. Blood.

[47] Mills EW, Green R, Ingolia NT (2017). Slowed decay of mRNAs enhances platelet specific translation. Blood.

