# Supplementary material for: Development and Validation of Tumor-educated Blood Platelets Integrin Alpha 2b (ITGA2B) RNA for Diagnosis and Prognosis of Non-small-cell Lung Cancer through RNA-seq
Source: Int J Biol Sci. 2019 Jul 24;15(9):1977–92. doi: 10.7150/ijbs.36284 (PMC6743295; doi:10.7150/ijbs.36284)
Supplement: Supplementary file 1 — Supplementary figures and tables. [file ijbsv15p1977s1.pdf]

**Supplementary materials**

**clinicopathologic characteristics of patients and controls in the discovery and screening group**

17 platelets from 9 NSCLC (8 stage I and 1 stage II; 6 ADC and 3 SqCC; ages 46-83 years; median = 60 years; 4 males and 5 females) and 8 healthy controls (ages 41-79 years; median = 58 years; 4 males and 4 females) were collected for RNA-seq. The preliminary screening group included 22 patients with NSCLC (5 stage I, 5 stage II, 7 stage III and 5 stage IV; 20 ADC and 2 SqCC; ages 47-78 years, median = 59 years, 14 males and 8 females), 10 patients with benign pulmonary nodules (BPN; 4 cases of inflammatory pseudotumor, 4 cases of tuberculosis and 2 cases of lung granulomatous; ages 44-70 years, median = 58 years, 7 males and 3 females ), and 15 HC (ages 46-75 years, median = 56 years, 9 males and 6 females)

**Assessment of platelet purity**

To assess sample purity, three freshly isolated and randomly selected platelet isolations in RNAlater were fixed in 3.7% paraformaldehyde and counted by the Sysmex XN2000 haematology analyser and stained by Wright-Gimsa. The platelet morphology was confirmed on a light microscope by two observers (Xing S and Zeng T), which displayed full of the vision with 0-1 other cell (Figure S1A, 200×；Figure S1B, 400×). Total platelet and nucleated cell counts was determined by the Sysmex XN2000 haematology analyser in both the sheath flow DC detection(Figure S1C) and PLT-fluorescent method (Figure S1D) and yielded an estimated 1 to 5 nucleated cell counts per 10 million platelets, which is in concordance to observations by others[1].

**The primers of the candidate TEP mRNA**

The following primers were used: BSG, forward primer: 5′-CAGCGGTTGGAGGTTGTAG -3′; reverse primer: 5′-GTGCCCTGTGACCTCTGTG -3′; CD63, forward primer: 5′-GGAAGGAGGAATGAAATGTG -3′; reverse primer: 5′-

CACTGCGATGATGACCACT -3′; DERA, forward primer: 5′-AGCCGCCGTTTGTGTTTAT -3′; reverse primer: 5′-ACGTCGATTTCTGTAGCTCCAT-3′; IFITM3, forward primer: 5′-GCCGCTGGTCTTCGCTG -3′; reverse primer: 5′-TCTTCCTGTCCCTAGACTTCACG-3′; TGFB1I1, forward primer: 5′-GCAAGGGCAGCCTAGACACC -3′; reverse primer: 5′-ACAGCCTCCGCAAACGAAG -3′; TIMP1, forward primer: 5′-GCTTCTGGCATCCTGTTGTTG-3′; reverse primer: 5′-TAACGCTGGTATAAGGTGGTCTG-3′; TLN1, forward primer: 5′-TGACATCCTGAATGGCTCC -3′; reverse primer: 5′-CCCTTCTGCTTCACATACTCC -3′; and TPM1, forward primer: 5′-GCCGACGTAGCTTCTCTGAAC -3′; reverse primer: 5′-TTTGGGCTCGACTCTCAATGAC -3′.

**The comparison of traditional tumor markers, CEA and CYFRA21-1 in NSCLC diagnosis**

we assessed and compared CEA, and CYFRA21-1 in a 127 NSCLC and 62 controls of test cohort, and found that CEA performed better than CYFRA21-1 in the diagnosis of NSCLC, with an AUC of 0.775 (95% CI, 0.710 to 0.840) and an AUC of 0.689 (95% CI, 0.612 to 0.766), respectively, shown in the Figure S2.

**The protein expression levels of platelet ITGA2B and SELP**

We detected the protein expression levels of platelet ITGA2B and SELP. The antibodies for Flow Cytometry (FC) and western blotting (WB) was purchased from R&D system, USA. FC was performed using a BD FACSCalibur flow cytometer. All the experiments were done following the manufacturer’s instructions. As shown in the Figure S4, FC results showed that no difference between NSCLC group and HC

group was found. We also conducted western blotting. In brief, platelet proteins were separated by sodium dodecyl sulfate polyacrylamide gel electrophoresis and transferred to polyvinylidene difluoride membranes. Then, they are incubated with antibodies to ITGA2B (2ug/ml, MAB7616, R&D), SELP (1ug/ml, AF137, R&D), and α-tubulin (1:3,000, ab9781, Abcam) at 4°C overnight, then treated with a horseradish peroxidase-conjugated secondary antibody. The results were similar to FC.

**The expression and diagnostic analysis of PLT, MPV and IPF**

We utilized whole blood samples from the test cohort and validation cohort to estimate the discriminatory capability of PLT and MPV. 18 patients of NSCLC and 11 healthy controls were also introduced to evaluate the diagnostic effect of IPF absolute value (IPF#) and IPF fraction (IPF%). As shown in the Figure S5, the AUC values for PLT and MPV in discriminating the NSCLC patients from the controls were 0.562 (95% CI, 0.501 to 0.623), and 0.447 (0.368 to 0.509) in the test cohort, 0.419 (0.333 to 0.505) and 0.471 (0.385 to 0.557) in the validation cohort, respectively. Moreover, neither the IPF# nor IPF% had good diagnostic performance, with an AUC of 0.447 (95% CI, 0.226 to 0.668) and an AUC of 0.510 (95% CI, 0.284 to 0.736), respectively.

**The diagnostic performance of platelet ITGA2B in subgroups of NSCLC**

CEA is the most common used tumor markers in NSCLC. However, its sensitivity is low, especially at the stage I stage. We tried to evaluate whether ITGA2B had supplementary diagnostic value for this CEA negative subgroup of NSCLC. ROC curves were plotted for platelet ITGA2B in those CEA-negative NSCLC patients versus different control groups, seen in the Figure S6. In the detection of CEA-negative NSCLC patients from all control subjects (HC and BPN), the AUC of ITGA2B was 0.951 (95% CI 0.907-0.996) with a sensitivity of 97.8% and specificity of 78.6%. In the differentiation of CEA-negative NSCLC from the BPN, the similar result was found. In addition, the diagnostic values of platelet ITGA2B in CEA-positive NSCLC were also investigated. The ROC curves shown in Figure S6 E-H indicated that platelet ITGA2B could distinguish CEA-positive NSCLC from

noncancerous population. Simultaneously, the validation cohort verified the above diagnostic significance of platelet ITGA2B.

**The diagnostic effect of different combination of platelet ITGA2B, platelet SELP and serum CEA**

ROC analysis showed that the diagnostic accuracy for NSCLC in the test cohort when all the three markers were tested was similar to the combination of ITGA2B and CEA (AUC: 0.957 vs 0.957; sensitivity: 90.1% vs 90.1%; and specificity: 86.4% vs 86.9%), whereas was superior to ITGA2B and SELP combination (AUC: 0.929; sensitivity: 88.8%; and specificity: 82.5%), or SELP plus CEA (AUC: 0.801; sensitivity: 71.7%; and specificity: 76.2%), the results were similar in the validation cohort, seen in the Table S4 and Figure S7.

**The diagnostic performance of platelet ITGA2B in subgroups of stage I NSCLC**

Furthermore, the diagnostic values of platelet ITGA2B in the subgroup of CEA-negative stage I NSCLC have been explored. The ROC curves were also plotted for ITGA2B in CEA-negative stage I NSCLC versus different control groups, seen in the Figure S8. In detection of CEA-negative stage I-NSCLC from all control subjects (HC and BPN), the AUC of ITGA2B was 0.938 (95% CI 0.861-1.000) with a sensitivity of 96.0% and specificity of 78.6%. In differentiation of CEA-negative stage I-NSCLC from BPN, the sensitivity was the same (96.0%) with a specificity of 81.7%. The ROC curves in Figure S8E, 8G showed that platelet ITGA2B mRNA could discriminate CEA-positive stage I NSCLC from noncancerous people. Similar results were obtained in the validation cohort.

**The diagnostic performance of platelet SELP in validation cohort**

In detection of NSCLC from all control subjects (HC and BPN), the AUC of SELP was 0.716 (95% CI 0. 616-0.815, Figure S9) with a sensitivity of 96.7% and specificity of 43.1%. In differentiation of NSCLC from BPN, the sensitivity was the same (96.7%) with a specificity of 43.8%. In the differentiation of stage I NSCLC from the BPN, the similar result was found.

**The diagnostic performance of platelet ITGA2B in ADC or SqCC**

As shown in Figure S10, the values of platelet ITGA2B did not differ significantly between the two groups in both cohorts. ROC curves analyses illustrated that the levels of platelet ITGA2B mRNA were robust in discriminating ADC from controls, with an AUC value of 0.925 (0.894-0.956), a sensitivity of 93.4% and a specificity of 78.6% in the test cohort and 0.878 (0.823-0.933), 75.7% and 89.4% in the validation group, and also SqCC from controls, with an AUC value of 0.895 (0.811-0.980), a sensitivity of 86.7% and a specificity of 86.9% in the test cohort and 0.930 (0.811-0.979), 100.0% and 81.2% in the validation group.

# References:


1. Rolf N, Knoefler R, Suttorp M, Kluter H, Bugert P. Optimized procedure for platelet RNA profiling from blood samples with limited platelet numbers. Clin Chem. 2005; 51: 1078-80.


Table S1 Steps in refining the biomarker candidates

| TEPs | refinement steps | Figure |
|---|---|---|
| 1220 up and 570 down | number of DEGs | Figure 2A |
| 208 overlappped | reproducibilitity in GSE68086 and 89843 | Figure 2D |
| 148 upregulated | upregulated DEGs | |
| 119 upregulated in at least 7/ 9 NSCLC | sensitivity | |
| 104 RPKM > 20 | easier to be detected | |
| 8 core DEGs | functionnally and previous report | Figure2B, C and E |
| 2 TEP | validated by q-PCR | Figure S2 |

TEPs:tumor educated platelets; DEGs: differentially expressed genes

**Table S2 Expression level of platelet ITGA2B, SELP and serum CEA in different groups in both test and validation cohorts**

| Group | Test | | | Validation | | |
|---|---|---|---|---|---|---|
| | No | Median(IQR) | Mean(SD) | No | Median(IQR) | Mean(SD) |
| **ITGA2B** | | | | | | |
| **HC** | 97 | 0.000357 (0.00005-0.001941) | 0.004106(0.011734) | 53 | 0.005441 (0.001112-0.067296) | 0.005097(0.010402) |
| **BPN** | 109 | 0.000243 (0.000108-0.000694) | 0.003609(0.012148) | 32 | 0.005392 (0.000405-0.283876) | 0.021366(0.062388) |
| **NSCLC** | 152 | 0.048202 (0.014353-0.111214) | 0.227023(0.939929) | 91 | 0.310644 (0.043569-8.339712) | 0.516706(1.349308) |
| **Stage I-NSCLC** | 56 | 0.0720216 (0.024313-0.124673) | 0.082197(0.071806) | 41 | 0.061039 (0.029022-3.08086) | 0.197329(0.618815) |
| **SELP** | | | | | | |
| **HC** | 97 | 0.001413 (0.000368-0.003164) | 0.004896(0.01713) | 35 | 0.004888 (0.000635-0.032203) | 0.0290246(0.051443) |
| **BPN** | 109 | 0.001767 (0.000815-0.00329) | 0.011983(0.058061) | 16 | 0.004492 (0.001205-0.01052) | 0.006182(0.005294) |
| **NSCLC** | 152 | 0.01059 (0.003362-0.00329) | 0.075791(0.395953) | 61 | 0.016941 (0.00861-0.040768) | 0.0645336(0.14448) |
| **Stage I-NSCLC** | 56 | 0.012845 (0.004982-0.026132) | 0.095027(0.573461) | 29 | 0.011385 (0.007290.021002-) | 0.023628(0.035399) |
| **CEA** | | | | | | |
| **HC** | 97 | 1.97(1.235-2.765) | 2.27367(1.488934) | 53 | 1.61(1.01-2.75) | 1.967849(1.271853) |
| **BPN** | 109 | 1.71(1.045-2.98) | 2.17006(1.479845) | 32 | 1.575(1.075-2.2925) | 1.851219(0.94282) |

| | | | | | | |
|---|---|---|---|---|---|---|
| **NSCLC** | 152 | 3.995(1.045-9.2975) | 24.4346(84.011607) | 91 | 4.14(1.9-10.22) | 17.55122(52.309013) |
| **Stage I-NSCLC** | 56 | 3.15(1.825-5.0425) | 5.42854(8.299524) | 41 | 3.06(1.5-4.9) | 7.921976(21.714067) |

NSCLC: non-small cell lung cancer; BPN: benign pulmonary nodules; HC: healthy controls; IQR: interquartile range; SD: standard definition; HC: healthy controls; ITGA2B: Integrin, Alpha 2b; SELP: P-selectin; CEA: carcinoembryonic antigen.

Table S3 Results for measurement of platelets ITGA2B mRNA in the diagnosis of CEA-negative or CEA-positive patients with NSCLC

| | Test | | | | | | | Validation | | | | | | |
|---|---|---|---|---|---|---|---|---|---|---|---|---|---|---|
| | AUC(95%CI) | SN (%) | SP (%) | PPV (%) | NPV (%) | Positive LR | Negative LR | AUC(95%CI) | SN (%) | SP (%) | PPV (%) | NPV (%) | Positive LR | Negative LR |
| **CEA negative** | | | | | | | | | | | | | | |
| NSCLC vs BPN and HC | 0.951(0.907-0.996) | 97.8 | 78.6 | 50.6 | 99.4 | 4.57 | 0.03 | 0.905(0.847-0.963) | 94.6 | 56.5 | 48.6 | 96.0 | 2.17 | 0.10 |
| NSCLC vs BPN | 0.952(0.906-0.998） | 97.8 | 81.7 | 69.2 | 98.9 | 5.34 | 0.03 | 0.883(0.798-0.969) | 94.6 | 59.4 | 72.9 | 90.5 | 2.33 | 0.09 |
| Stage I NSCLC vs BPN and HC | 0.938(0.861-1.000) | 96.0 | 78.6 | 35.3 | 99.4 | 4.49 | 0.05 | 0.882(0.803-0.962) | 95.0 | 56.5 | 33.9 | 98.0 | 2.18 | 0.09 |
| Stage I NSCLC vs BPN | 0.939(0.861-1.000) | 96.0 | 81.7 | 54.5 | 98.9 | 5.25 | 0.05 | 0.861(0.758-0.964) | 95.0 | 59.4 | 59.4 | 95.0 | 2.34 | 0.08 |
| **CEA positive** | | | | | | | | | | | | | | |
| NSCLC vs BPN and HC | 0.909(0.873-0.946) | 90.6 | 78.6 | 68.6 | 94.2 | 4.23 | 0.12 | 0.876(0.814-0.939) | 88.9 | 56.5 | 56.5 | 88.9 | 2.04 | 0.20 |
| NSCLC vs BPN | 0.916(0.876-0.955） | 90.6 | 81.7 | 82.8 | 89.9 | 4.95 | 0.12 | 0.862(0.779-0.946) | 88.9 | 59.4 | 78.7 | 76.0 | 2.19 | 0.19 |
| Stage I NSCLC vs BPN and HC | 0.941(0.878-1.000） | 96.8 | 78.6 | 40.5 | 99.4 | 4.52 | 0.04 | 0.803(0.697-0.909） | 81.0 | 56.5 | 31.5 | 92.3 | 1.86 | 0.34 |
| Stage I NSCLC vs BPN | 0.942(0.87 | 96.8 | 81.7 | 60.0 | 98.9 | 5.29 | 0.04 | 0.792(0.668-0. | 81.0 | 59.4 | 56.7 | 82.6 | 2.00 | 0.32 |

| | | | |
|---|---|---|---|
| | 7-1.000 ) | | 915 ) |

AUC: area under curve; SN: sensitivity; SP:specificity; PPV: positive predictive value; NPV: negative predictive value; NSCLC: non-small cell lung cancer; BPN: benign pulmonary nodules; HC: healthy controls; ITGA2B: Integrin, Alpha 2b; CEA: carcinoembryonic antigen.

Table S4 Results for combination of platelets ITGA2B, SELP mRNA, and serum CEA in the diagnosis of NSCLC versus controls in both cohorts

| Variable | AUC(95%CI) | Cutoff | Sensitivity (%) | Specificity (%) | PPV (%) | NPV (%) | Positive LR | Negative LR |
|---|---|---|---|---|---|---|---|---|
| Test cohort | | | | | | | | |
| ITGA2B+CEA | 0.957(0.939-0.975) | 0.29188 | 90.1 | 86.9 | 83.5 | 92.3 | 6.88 | 0.11 |
| ITGA2B+SELP | 0.929(0.901-0.956) | 0.19309 | 88.8 | 82.5 | 78.9 | 90.9 | 5.07 | 0.14 |
| ITGA2B+SELP+CEA | 0.957(0.939-0.976) | 0.27688 | 90.1 | 86.4 | 83 | 92.2 | 6.63 | 0.11 |
| SELP+CEA | 0.801(0.754-0.848) | 0.34910 | 71.7 | 76.2 | 69 | 78.5 | 3.01 | 0.37 |
| Validation cohort* | | | | | | | | |
| ITGA2B+CEA | 0.908(0.854-0.961) | 0.42836 | 86.9 | 84.3 | 86.9 | 84.3 | 5.54 | 0.16 |
| ITGA2B+SELP | 0.846(0.774-0.918) | 0.42995 | 72.1 | 90.2 | 89.8 | 73 | 7.36 | 0.31 |
| ITGA2B+SELP+CEA | 0.899(0.844-0.955) | 0.35033 | 86.9 | 80.4 | 84.1 | 83.7 | 4.43 | 0.16 |
| SELP+CEA | 0.821(0.744-0.897) | 0.55169 | 65.6 | 88.2 | 86.9 | 68.2 | 5.56 | 0.39 |

AUC: area under curve; PPV: positive predictive value; NPV: negative predictive value; NSCLC: non-small cell lung cancer; ITGA2B: Integrin, Alpha 2b; SELP: P-selectin; CEA: carcinoembryonic antigen.

*in validation cohort, a subgroup of the subjects, that is, 61 NSCLC and 51controls were measured SELP.

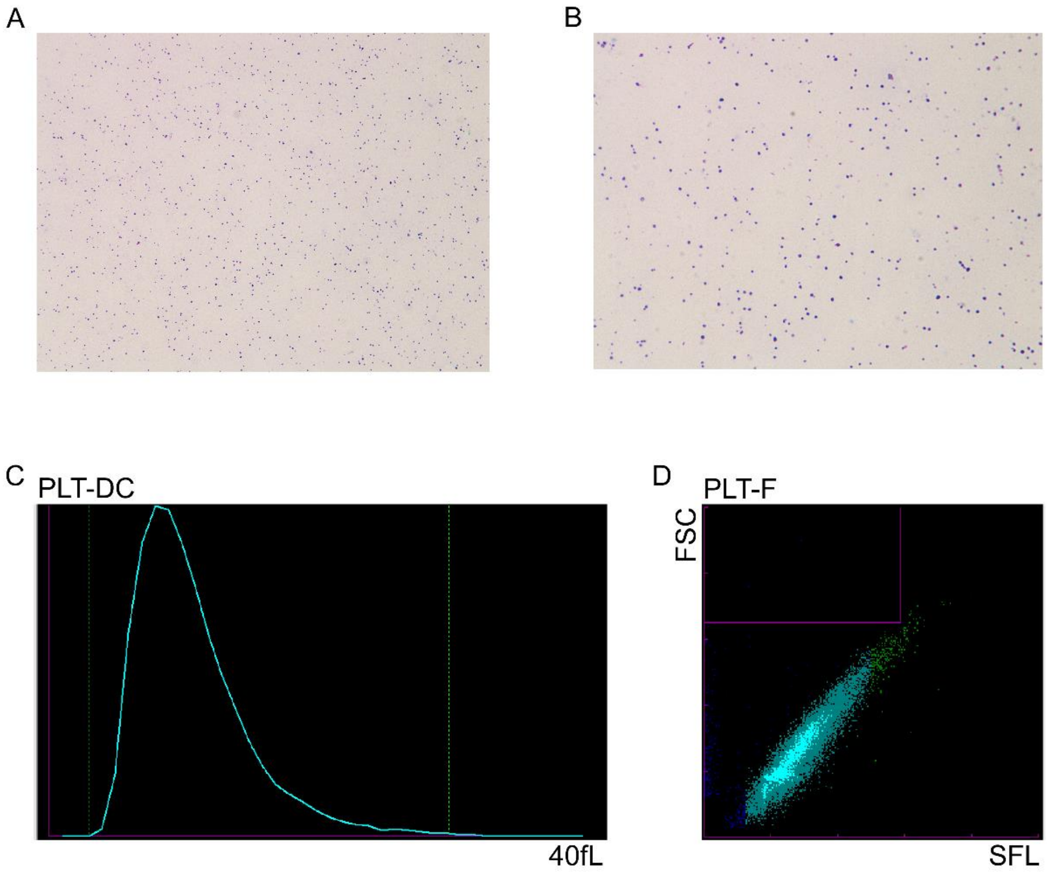


**Figure S1. Platelet purify and quantity**

Representative Wright-Gimsa staining of platelet (A, 200× and B, 400×). Total platelet counts in both the sheath flow DC detection (C) and PLT-fluorescent method (D) PLT-DC: platelet direct current; PLT-F: PLT-fluorescent.

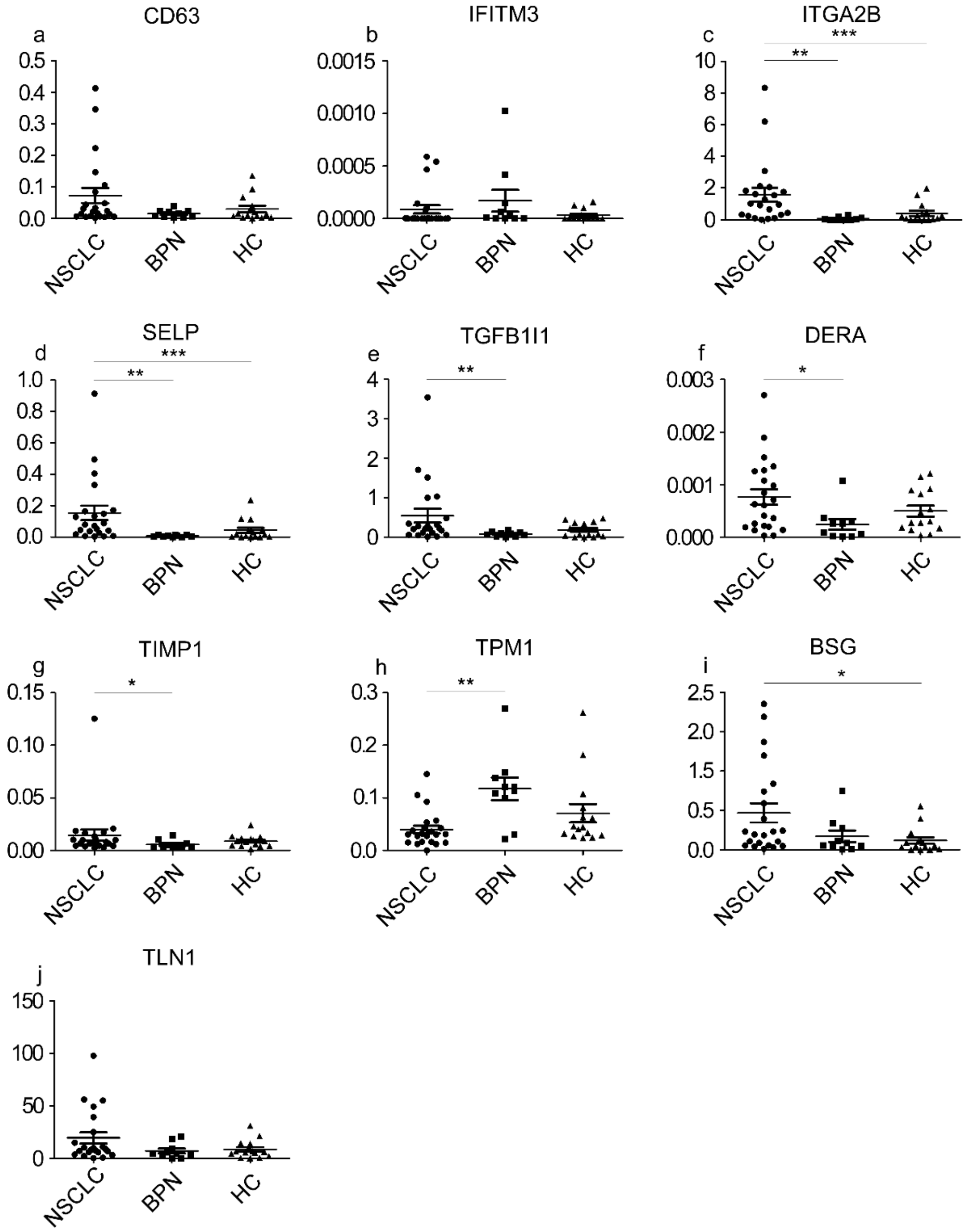


**Figure S2. Levels of 8 candidate platelet mRNA and 2 negative controls in the preliminary screening phase**

Levels of platelets BSG, CD63, DERA, IFITM3, ITGA2B, SELP, TGFB1I1, TIMP1, TLN1, and TPM1 were compared between 22 patients with NSCLC, 10 patients with benign pulmonary nodules and 15 healthy controls. The Mann-Whitney U test was performed for comparisons between groups. $p < 0.05$ was considered statistically significant.

NSCLC: non-small cell lung cancer; BPN: benign pulmonary nodules; HC: healthy controls

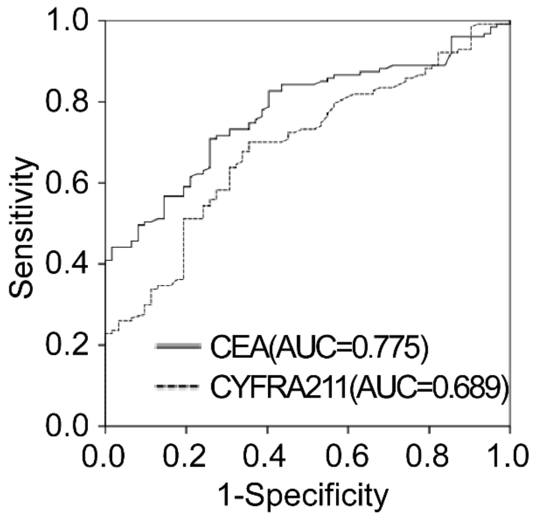


**Figure S3. Diagnostic outcomes for CEA and CYFRA21-1 in the NSCLC**

CEA: carcinoembryonic antigen; CYFRA21-1: Cytokeratin 19; AUC: areas under the curves

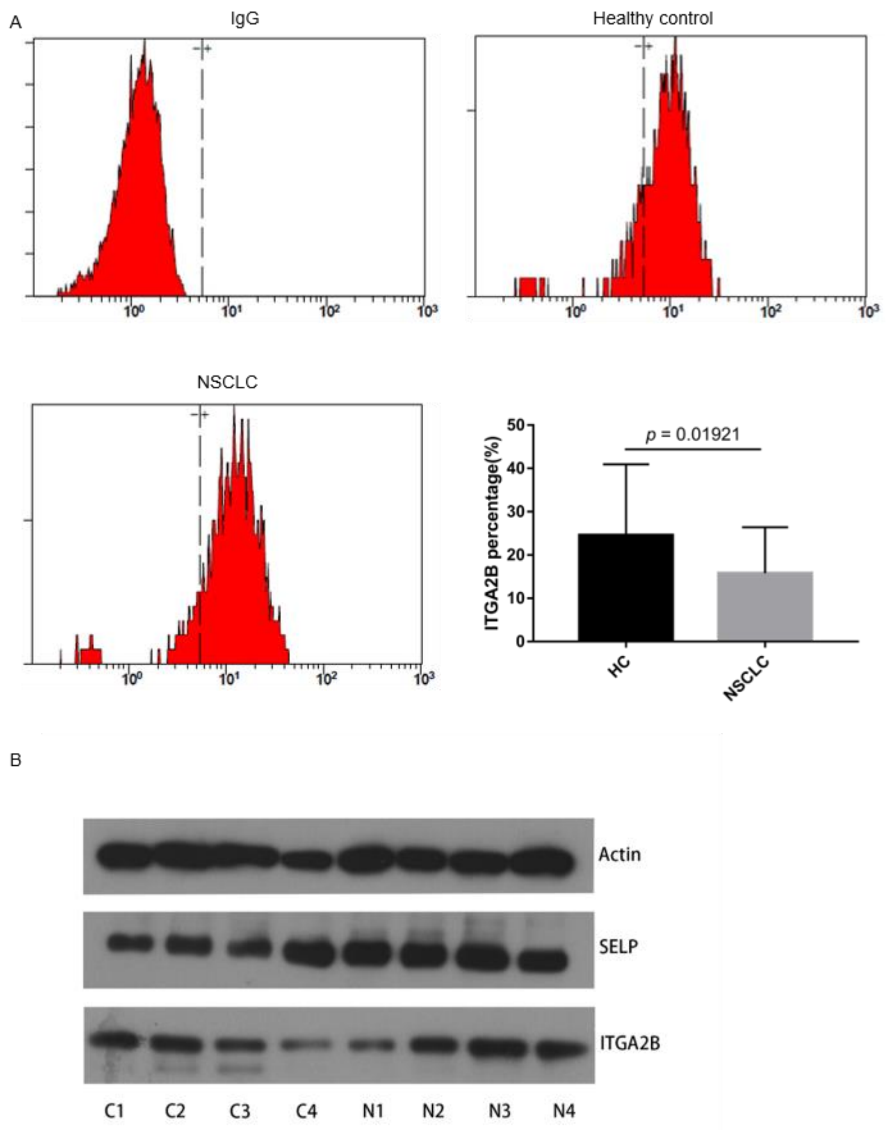


**Figure S4. Levels of platelet ITGA2B and SELP protein**

(A) Representative histogram of platelet ITGA2B in healthy control and NSCLC. The MFI from 5 HC and 13 NSCLC was analyzed by Mann-Whitney U test.

(B) Western blotting of platelet ITGA2B and SELP in 4 HC and 4 NSCLC. NSCLC: non-small cell lung cancer; HC: healthy controls; MFI: mean fluorescence intensity.

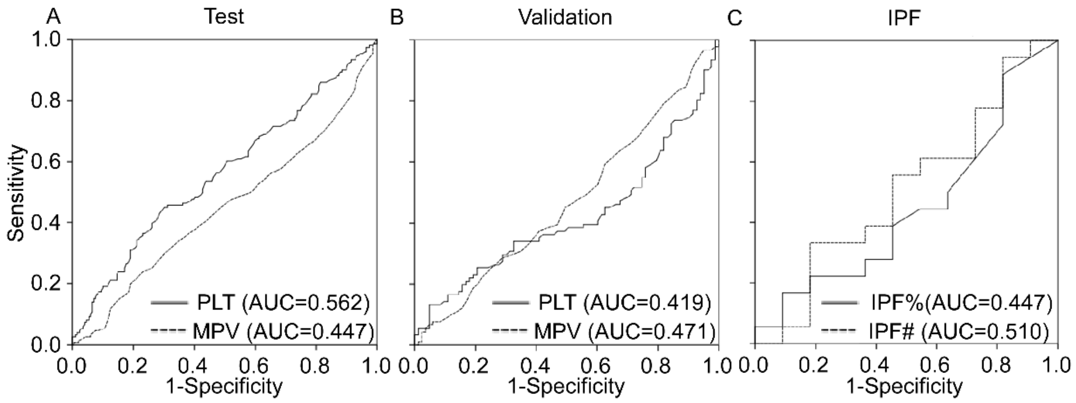


**Figure S5. Diagnostic outcomes for platelet counts (PLT), mean platelet volume (MPV) and immature platelet fraction (IPF) in the NSCLC**

(A) ROC curve for PLT, and MPV with NSCLC versus all controls in the test cohort. (B) ROC curve for PLT, and MPV with NSCLC versus all controls in the validation cohort. (C) ROC curve for IPF#, and IPF% with 18 NSCLC versus 11 healthy controls.

PLT: platelet counts; MPV: mean platelet volume; IPF#: immature platelet fraction absolute value and IPF%: immature platelet fraction fraction; ROC: receiver operating characteristics; AUC: areas under the curves.

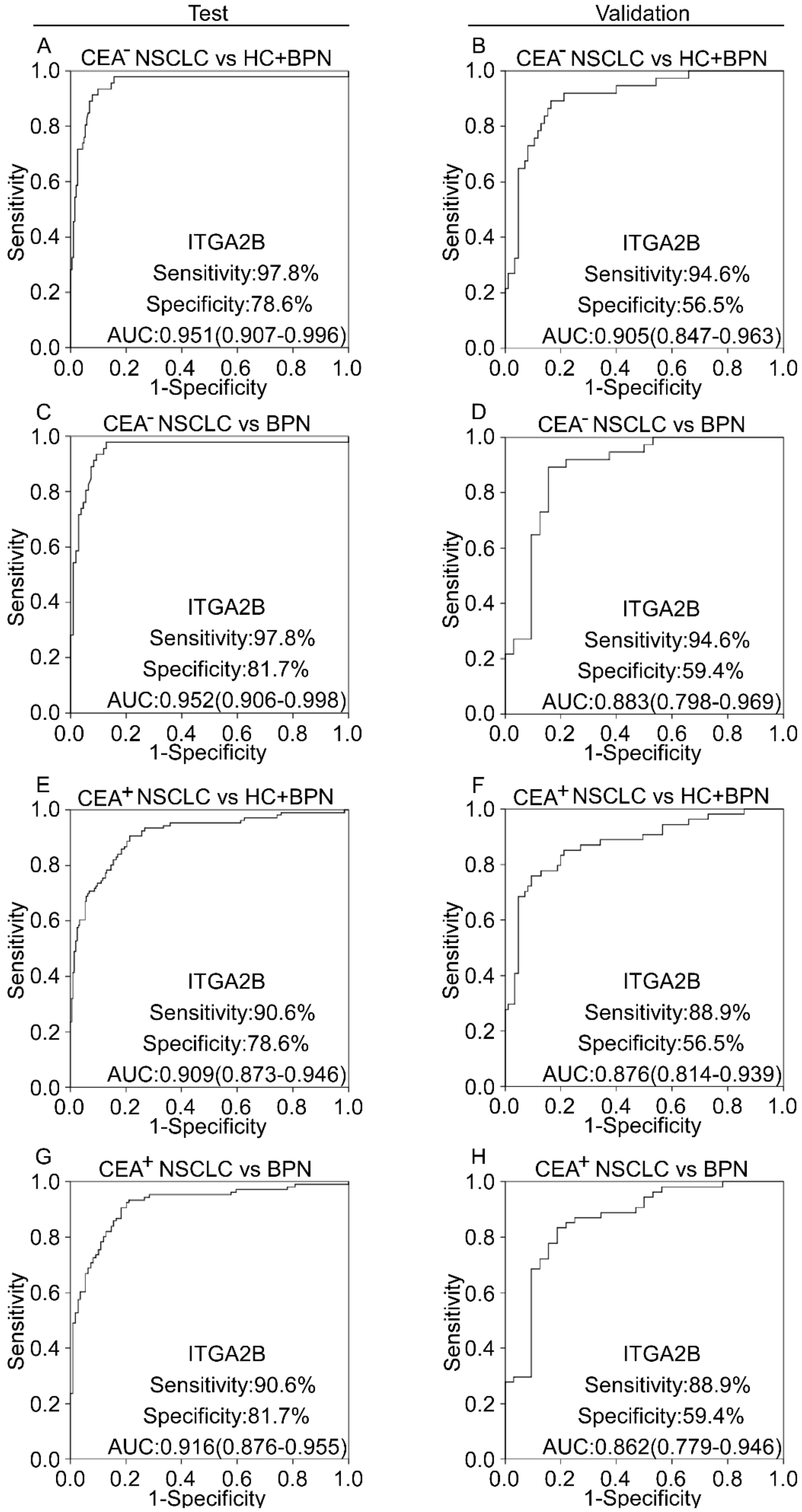
Test
Validation
A
CEA⁻ NSCLC vs HC+BPN
ITGA2B
Sensitivity:97.8%
Specificity:78.6%
AUC:0.951(0.907-0.996)
B
CEA⁻ NSCLC vs HC+BPN
ITGA2B
Sensitivity:94.6%
Specificity:56.5%
AUC:0.905(0.847-0.963)
C
CEA⁻ NSCLC vs BPN
ITGA2B
Sensitivity:97.8%
Specificity:81.7%
AUC:0.952(0.906-0.998)
D
CEA⁻ NSCLC vs BPN
ITGA2B
Sensitivity:94.6%
Specificity:59.4%
AUC:0.883(0.798-0.969)
E
CEA+ NSCLC vs HC+BPN
ITGA2B
Sensitivity:90.6%
Specificity:78.6%
AUC:0.909(0.873-0.946)
F
CEA+ NSCLC vs HC+BPN
ITGA2B
Sensitivity:88.9%
Specificity:56.5%
AUC:0.876(0.814-0.939)
G
CEA+ NSCLC vs BPN
ITGA2B
Sensitivity:90.6%
Specificity:81.7%
AUC:0.916(0.876-0.955)
H
CEA+ NSCLC vs BPN
ITGA2B
Sensitivity:88.9%
Specificity:59.4%
AUC:0.862(0.779-0.946)
Sensitivity
1-Specificity
0.0
0.2
0.4
0.6
0.8
1.0

**Figure S6. Diagnostic capability of platelets ITGA2B in subgroups of NSCLC**

(A and B) ROC curve of ITGA2B in CEA-negative NSCLC patients versus all control subjects.

(C and D) ROC curve of ITGA2B in CEA-negative NSCLC patients versus BPN.

(E and F) ROC curve of ITGA2B in CEA -positive NSCLC patients versus all control subjects

(G and H) ROC curve of ITGA2B in CEA -positive NSCLC patients versus BPN.

NSCLC: non-small cell lung cancer; BPN: benign pulmonary nodules; HC: healthy controls; $CEA^-$: patients with negative CEA (serum CEA≤2.865 ng/ml). $CEA^+$: patients with positive CEA (serum CEA>2.865 ng/ml); ROC: receiver operating characteristics. AUC: areas under the curves. 95% CI: 95% confidence interval; ITGA2B: Integrin, Alpha 2b; CEA: carcinoembryonic antigen.

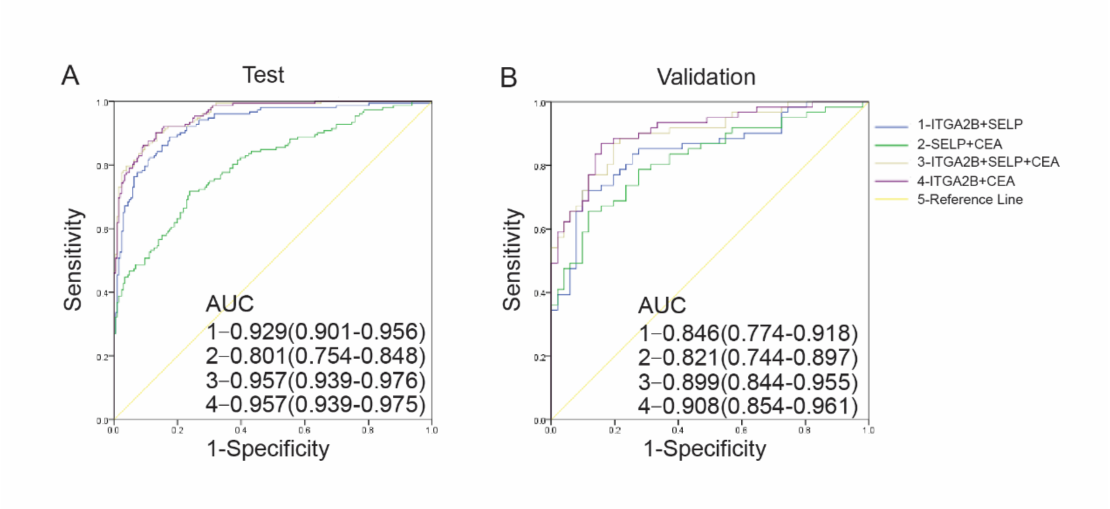


**Figure S7. Comparison of diagnostic capability of the combination of ITGA2B and CEA, ITGA2B and SELP, ITGA2B, CEA and SELP, SELP and CEA in the test(A) and validation cohort(B).**

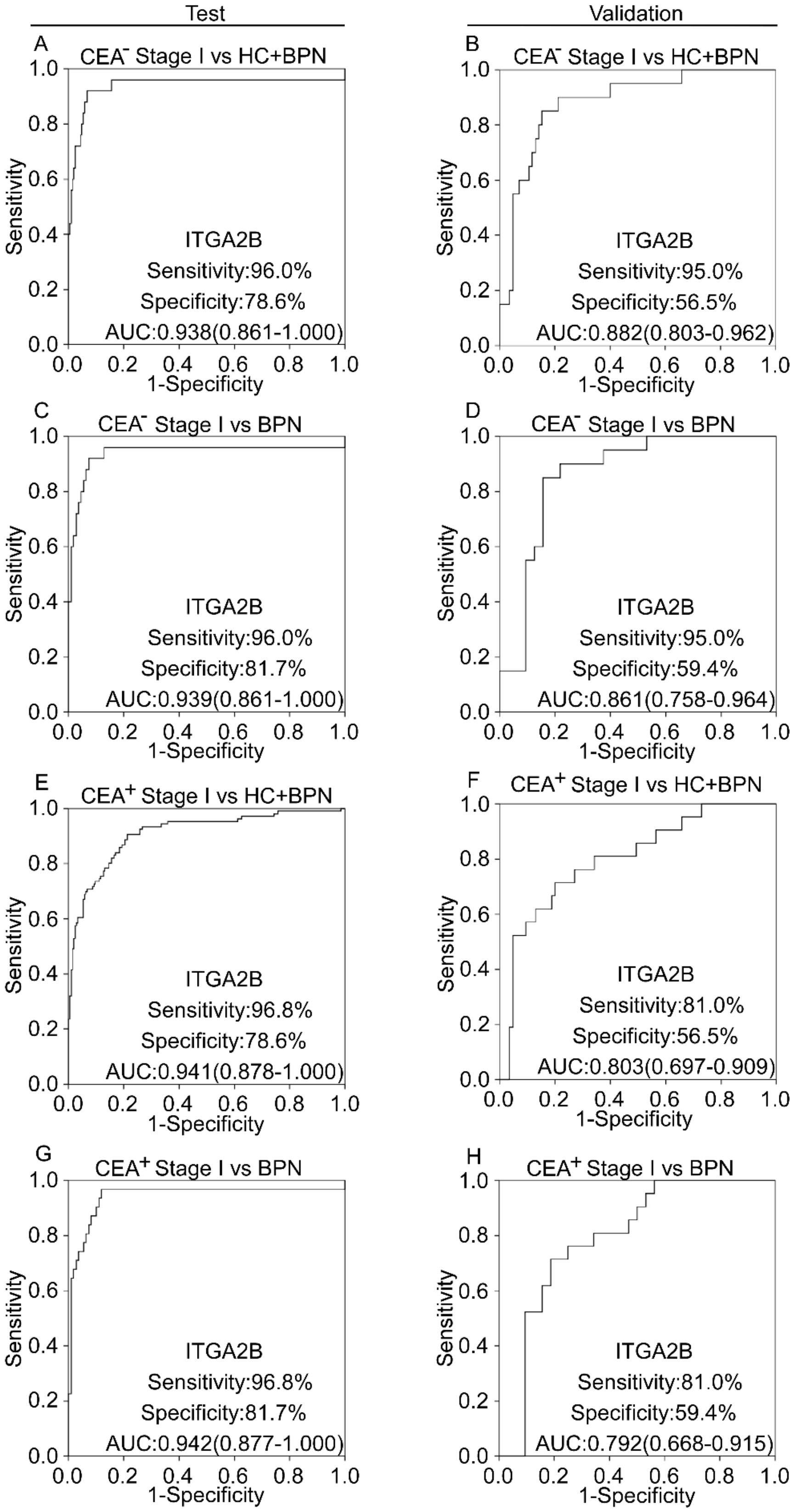


**Figure S8. Diagnostic capability of platelets ITGA2B in subgroups of stage I NSCLC**

(A and B) ROC curve of ITGA2B in CEA-negative stage I NSCLC patients versus all control subjects.

(C and D) ROC curve of ITGA2B in CEA-negative stage I NSCLC patients versus BPN.

(E and F) ROC curve of ITGA2B in CEA -positive stage I NSCLC patients versus all control subjects

(G and H) ROC curve of ITGA2B in CEA -positive stage I NSCLC patients versus BPN.

NSCLC: non-small cell lung cancer; BPN: benign pulmonary nodules; HC: healthy controls; $CEA^-$: patients with negative CEA (serum CEA ≤ 2.865 ng/ml); $CEA^+$: patients with positive CEA (serum CEA > 2.865 ng/ml); ROC: receiver operating characteristics; AUC: areas under the curves. 95% CI: 95% confidence interval; ITGA2B: Integrin, Alpha 2b; CEA: carcinoembryonic antigen.

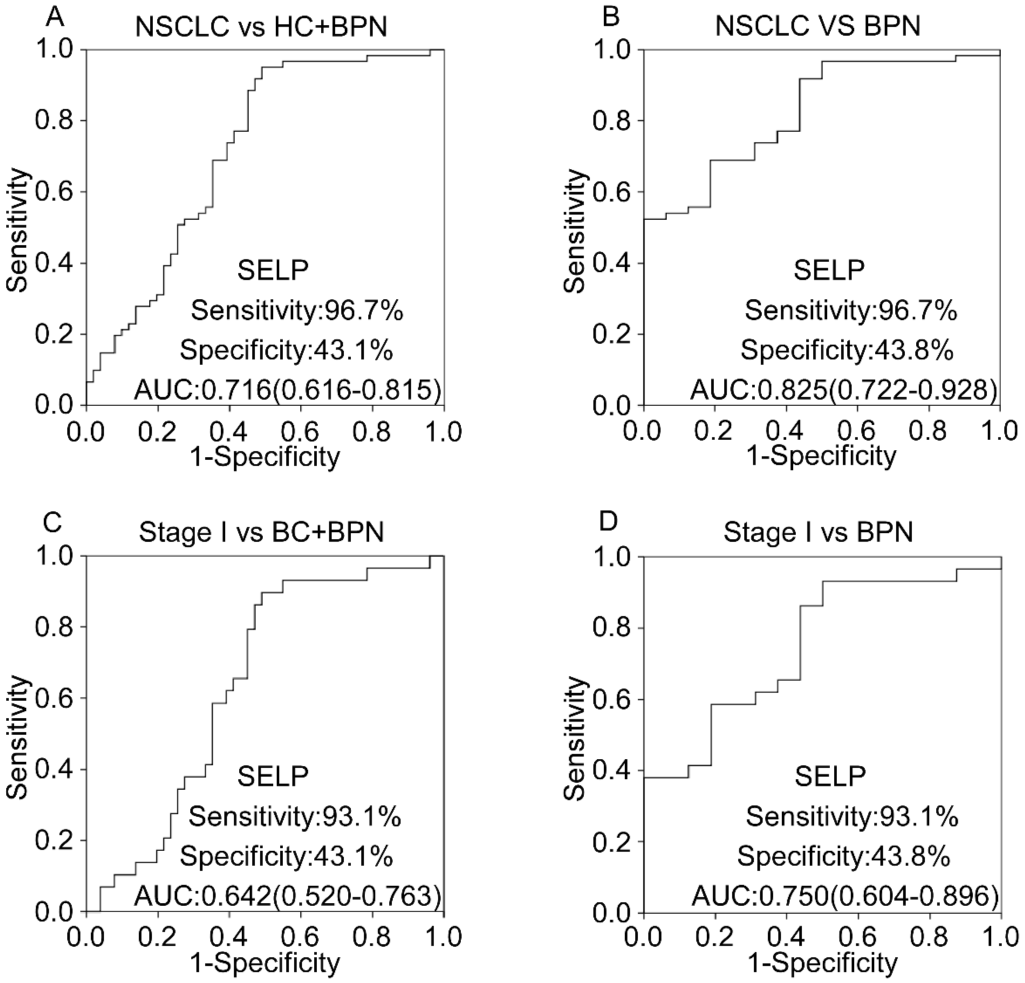


**Figure S9. Diagnostic outcomes for platelet SELP mRNA in the validation cohort**

(A) ROC curve for platelet SELP mRNA with NSCLC versus all controls. (B) ROC curve for platelet SELP mRNA with NSCLC versus BPN. (C) ROC curve for platelet SELP mRNA with stage I NSCLC versus all controls. (D) ROC curve for platelet SELP mRNA with stage I NSCLC versus BPN.

NSCLC: non-small cell lung cancer; BPN: benign pulmonary nodules; HC: healthy controls; SELP: P-selectin; ROC: receiver operating characteristics. AUC: areas under the curves. 95% CI: 95% confidence interval.

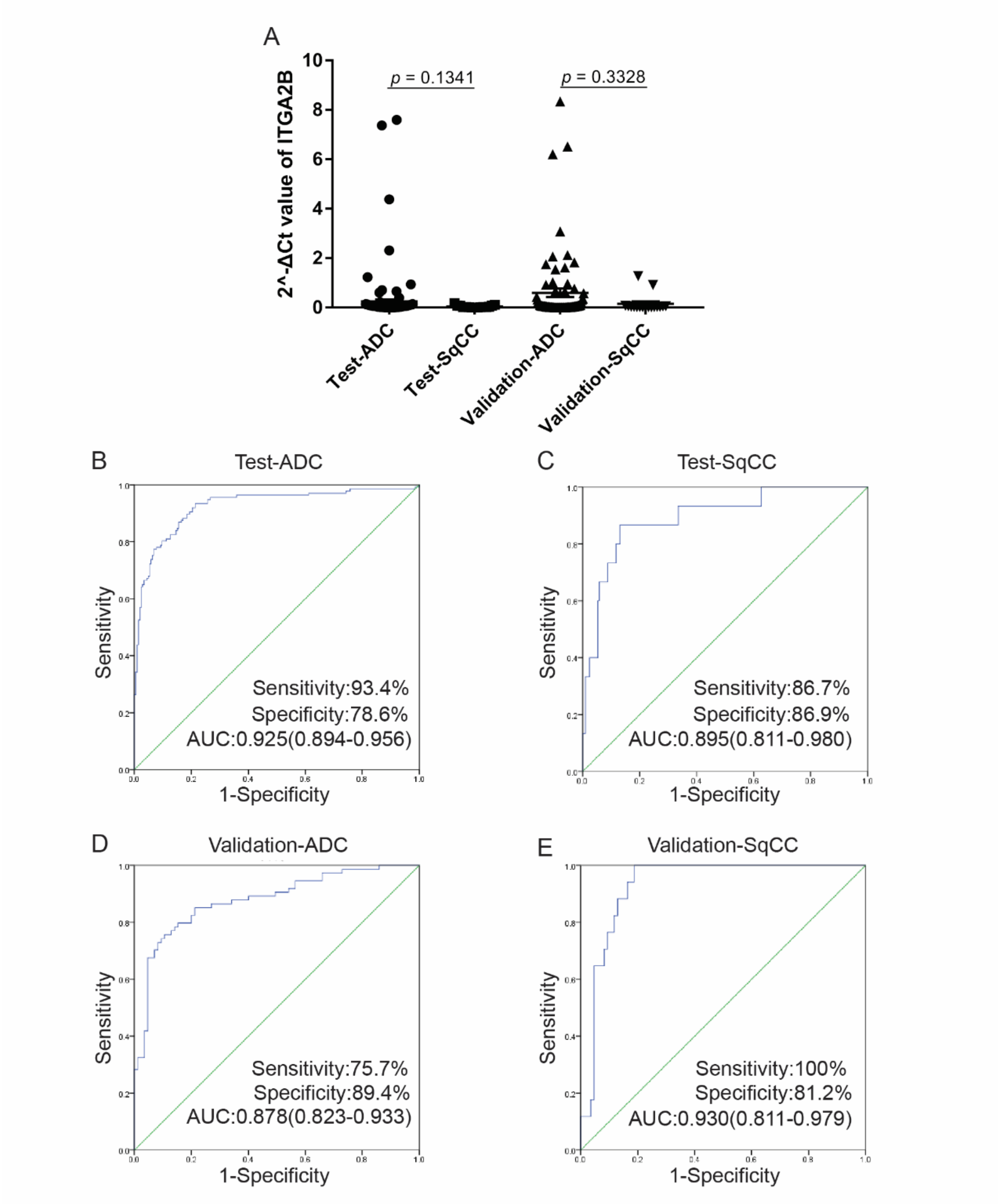


**Figure S10. Diagnostic outcomes for platelet ITGA2B mRNA in the adenocarcinoma (ADC) and squamous cell carcinoma (SqCC) cohort**

(A) Levels of platelet ITGA2B mRNA; ROC curves for ADC in the test cohort(B); SqCC in the test cohort(C); ADC in the validation cohort(D); SqCC in the validation cohort(E).
